# Tumor B‐cell infiltration in platinum‐treated advanced muscle‐invasive urothelial carcinoma

**DOI:** 10.1002/1878-0261.70276

**Published:** 2026-06-01

**Authors:** Konrad Stawiski, Júlia Perera‐Bel, Alejo Rodriguez‐Vida, Nuria Juanpere, Jihyun Lee, Daniel E. Michaud, Jennifer L. Guerriero, Kent W. Mouw, Aristotelis Bamias, Filipe L. F. Carvalho, Joaquim Bellmunt

**Affiliations:** ^1^ Department of Radiation Oncology Dana Farber Cancer Institute Boston MA USA; ^2^ Department of Biostatistics and Translational Medicine Medical University of Lodz Poland; ^3^ PSMAR Hospital del Mar Medical Research Institute (IMIM) Barcelona Spain; ^4^ Medical Oncology Department Hospital del Mar, CIBERONC Barcelona Spain; ^5^ Department of Urology Brigham and Women's Hospital Boston MA USA; ^6^ Division of Breast Surgery, Department of Surgery Brigham and Women's Hospital Boston MA USA; ^7^ Harvard Medical School Boston MA USA; ^8^ Department of Radiation Oncology Brigham and Women's Hospital Boston MA USA; ^9^ National and Kapodistrian University of Athens Greece; ^10^ Department of Medical Oncology Dana Farber Cancer Institute Boston MA USA

**Keywords:** B cell, platinum chemotherapy, tumor microenvironment, urothelial cancer

## Abstract

Platinum chemotherapy is a standard therapeutic choice for advanced urothelial cancer, but biomarkers demonstrating its benefits are limited. We tested whether the tumor microenvironment, particularly B‐cell infiltration, could predict overall survival after platinum chemotherapy. Pretreatment tumors from 189 patients in three cohorts treated with cisplatin‐ or carboplatin‐based regimens underwent bulk transcriptome profiling. Immune cell infiltration and multicellular communities were inferred from gene expression and associated with overall survival using cohort‐specific multivariable models and meta‐analysis. Higher lymphocyte infiltration was associated with longer overall survival (hazard ratio 0.34, 95% confidence interval 0.16–0.72), driven by B cells and memory B cells (hazard ratio 0.19, 95% confidence interval 0.05–0.75). These associations were confined to cisplatin‐treated patients and were strongest in tumors with a pro‐inflammatory B‐cell‐rich community and concordant B‐cell gene expression signatures. In contrast, higher myeloid infiltration was associated with shorter overall survival. Pretreatment B‐cell enrichment in the tumor microenvironment identified a subset associated with improved overall survival among patients receiving platinum chemotherapy, particularly cisplatin, supporting B‐cell‐focused biomarker development and spatial characterization of tertiary lymphoid structures in urothelial cancer.

## Graphical abstract

Bladder tumors with higher pretreatment memory B‐cell infiltration were linked to longer survival after cisplatin chemotherapy, but not carboplatin. These tumors also showed more organized immune structures (tertiary lymphoid structures) and a shared pro‐inflammatory B‐cell‐rich community, suggesting that memory B cells may help identify patients most likely to benefit from cisplatin.

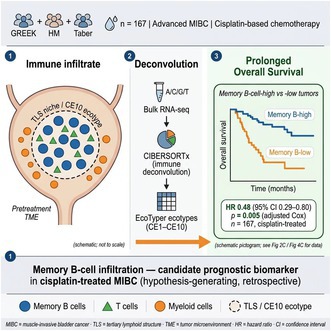

**Abbreviations**
CE: cellular ecotypes
CI: confidence interval
CR: complete response
DD‐GC: dose‐dense gemcitabine and cisplatin
DD‐MVAC: dose‐dense methotrexate, vinblastine, doxorubicin, and cisplatin
ECO: Eastern Cooperative Oncology Group
EV: enfortumab vedotin
FFPE: formalin‐fixed, paraffin‐embedded
HR: hazard ratio
IFN: interferon
MIBC: muscle‐invasive bladder cancer
MVAC: methotrexate, vinblastine, doxorubicin, and cisplatin
OS: overall survival
PR: partial response
RECIST: Response Evaluation Criteria in Solid Tumors
RIN: RNA integrity numbers
RNA‐seq: RNA sequencing
TIL: tumor‐infiltrating lymphocyte
TME: tumor microenvironment
TPM: transcripts per million
TURBT: transurethral resection of bladder tumor

## 1 Introduction

Platinum‐based chemotherapy followed by radical cystectomy has been the standard of care for muscle‐invasive bladder cancer (MIBC) for the past three decades [1]. Randomized clinical trials [2] and meta‐analyses [3] have shown that cisplatin‐based neoadjuvant chemotherapy regimens are associated with improved overall survival (OS) compared to surgery alone. Interestingly, recent evidence from the NIAGARA Phase III clinical trial combining perioperative chemotherapy with immunotherapy demonstrated improved OS compared to chemotherapy alone [4]. These results implicate the bladder tumor microenvironment (TME) as a key feature in the response to perioperative systemic therapy and underscore the importance of understanding the interaction between chemotherapy and immunotherapy in bladder cancer.

A recent secondary analysis of the IMvigor130 clinical trial, in which patients with locally advanced and metastatic urothelial carcinoma were treated with either cisplatin or carboplatin in combination with atezolizumab, found that pre‐existing adaptive immunity (evidence of an existing T cell‐mediated immune response) in the TME was associated with improved response to immune checkpoint blockade [5]. Moreover, this analysis demonstrated that cisplatin, but not carboplatin, can modulate the tumor immune microenvironment and increase the efficacy of immune checkpoint inhibitors in patients with metastatic urothelial cancer [5]. The interplay between cisplatin or carboplatin and immune cell infiltration, as well as the impact of TME modulation on OS in urothelial cancer, remains largely unexplored. Although first‐line platinum combination therapy is increasingly replaced by newer regimens such as enfortumab vedotin (EV) and pembrolizumab [6], the therapeutic sequence of cisplatin‐based chemotherapy followed by switch maintenance immunotherapy remains a standard of care in regions where EV plus pembrolizumab is not yet available [7, 8].

In this study, we conducted an analysis of the transcriptional profiles of pretreatment tumors from patients with locally advanced, surgically incurable, or metastatic bladder cancer who underwent treatment with either cisplatin‐ or carboplatin‐based chemotherapy alone. We evaluated the relationships between overall survival (OS) and immune cell populations and identified strong associations with tumor‐infiltrating B cells.

## 2 Materials and methods

### 2.1 Patient cohorts

We analyzed pretreatment bladder tumor tissue from a phase III clinical trial (GREEK cohort, ACTRN 12610000845033), real‐world bladder cancer patient cohort (HM cohort), and previously published results from a cohort of 300 bladder cancer patients [9] referred to as the TABER cohort, from which only patients with locally advanced and metastatic cancer (surgically incurable) were included in the analysis. Our study included patients with locally advanced, surgically unresectable, or metastatic urothelial bladder cancer who were treated with cisplatin‐ or carboplatin‐based chemotherapy.

All three cohorts comprised patients with locally advanced, surgically incurable, or metastatic urothelial carcinoma treated with first‐line platinum‐based chemotherapy in the palliative setting. No patients receiving neoadjuvant chemotherapy followed by radical cystectomy were included in the final analysis. Additionally, no patients received concurrent immunotherapy, targeted therapy, or other systemic anticancer agents during the study period. The heterogeneity across cohorts in terms of treatment protocols and patient populations is addressed in the Discussion.

Clinical data, including diagnostic staging, treatment details, and follow‐up information, were collected for all the cohorts. Biopsies from the HM and GREEK cohorts were processed for bulk RNA sequencing (RNA‐seq), and publicly available RNA‐seq data from the Taber cohort were included in the analysis.

The GREEK cohort included patients from the HR 16/03 trial (ACTRN12610000845033), a prospective, open‐label, randomized phase III study comparing two dose‐dense chemotherapy regimens: methotrexate, vinblastine, doxorubicin, and cisplatin (DD‐MVAC) versus gemcitabine and cisplatin (DD‐GC) in patients with inoperable, metastatic, or relapsed urothelial cancer. [10] Patients with good performance status (ECOG 0–1) were randomly assigned to receive either DD‐MVAC or DD‐GC. The median follow‐up was 52.1 months (range, 89 events). The study concluded that DD‐GC was not superior to DD‐MVAC but was better tolerated.

The HM cohort comprised a prospectively observed group of real‐world patients with locally advanced, surgically incurable, or metastatic urothelial bladder cancer diagnosed and treated at Hospital del Mar (Barcelona, Spain) between 2005 and 2010 with follow‐up until 2012. The patients in this cohort were primarily treated with platinum‐based chemotherapy.

From the Taber cohort, we included only patients who received first‐line chemotherapy upon the detection of locally advanced (T4b) or metastatic disease. In the original publication, cisplatin‐based chemotherapy was administered to approximately 98% of patients.

In all cohorts, pretreatment staging was based on baseline imaging and pathological assessment of transurethral resection of bladder tumor (TURBT) or radical cystectomy specimens. Treatment response was defined as complete response (CR) or partial response (PR) based on post‐treatment cross‐sectional imaging according to the RECIST 1.1 guidelines. Overall survival (OS) was defined as the time from muscle‐invasive bladder cancer (MIBC) diagnosis to death or the end of follow‐up. Clinical characteristics of the cisplatin‐treated subgroup are summarized in Table S1.

### 2.2 Ethics statement

The GREEK cohort was derived from the HR 16/03 clinical trial (ACTRN12610000845033), a prospective randomized phase III study conducted by the Hellenic Cooperative Oncology Group (HeCOG). The study was conducted in accordance with the Declaration of Helsinki. The study protocol received approval from the appropriate institutional and national review boards at all participating centers. Translational research on archived tumor tissue was approved by the Bioethics Committee of the Aristotle University of Thessaloniki (approval #5/6.7.2016). Patients were enrolled between November 2003 and January 2008 across 12 Greek centers. All patients provided written informed consent before enrollment in the study. The HM cohort study conformed to the standards set by the Declaration of Helsinki and was approved by the Clinical Research Ethics Committee of Parc de Salut Mar (CEIm‐Parc de Salut Mar), Hospital del Mar (Barcelona, Spain; approval #2013/5030/I). Samples were collected between 2005 and 2010 at Hospital del Mar, Barcelona, Spain. For the Taber cohort, the study was approved by relevant institutional review boards as described in the original publication [9].

### 2.3 Bulk RNA‐sequencing

Total RNA was extracted from formalin‐fixed, paraffin‐embedded (FFPE) bladder tumor samples using the RNeasy Mini Kit (Qiagen) according to the manufacturer's instructions. RNA quality was assessed using an Agilent 2100 Bioanalyzer. Given that FFPE‐derived RNA is typically degraded (RIN values are generally in the 2–3 range for FFPE tissue), library quality was evaluated based on fragment size distribution and DV200 metrics, and libraries meeting quality thresholds were selected for sequencing. Sequencing libraries were prepared using an Illumina TruSeq Stranded mRNA Library Prep Kit. Libraries were sequenced on an Illumina NextSeq 500 platform to generate paired‐end reads 75 bp in length.

Raw sequencing data were processed using the nf‐core/rnaseq pipeline (version 3.14.0; https://nf-co.re/rnaseq/3.14.0/) implemented with Nextflow for reproducibility and scalability [11]. Briefly, initial quality control of the raw reads was performed using FastQC. Adapter sequences and low‐quality bases were trimmed using Trim Galore!, which incorporates Cutadapt [12]. The cleaned reads were aligned to the human reference genome (GRCh38) using the STAR aligner [13]. Gene expression quantification was conducted using featureCounts [14]. Additionally, transcript‐level quantification was performed using Salmon [15] in quasi‐mapping mode. Differential gene expression analysis was performed using DESeq2 [16].

To harmonize gene expression data across cohorts, counts from the GREEK and HM cohorts were generated using the same nf‐core/rnaseq pipeline. For the Taber cohort, published count‐level data were obtained and integrated with the proprietary cohorts. The combined expression matrix was normalized using trimmed mean of M‐values (TMM) normalization via edgeR [17] to account for differences in library size and RNA composition across samples and cohorts.

### 2.4 Immune cell infiltrates and cellular communities

**Fig. 1:**
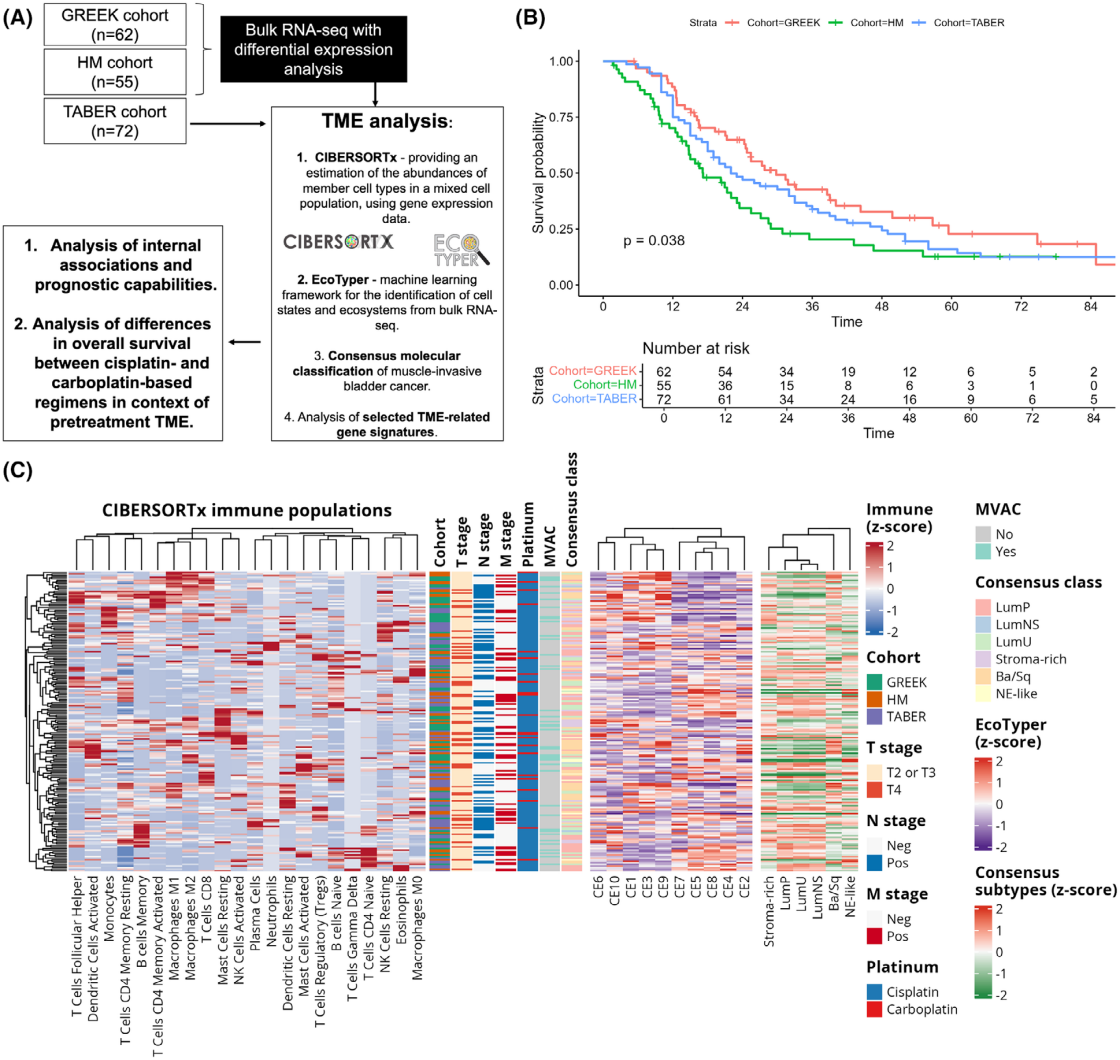
Study design, methodology, and cohort characteristics. (A) Overview of the study workflow. (B) Kaplan–Meier analysis of overall survival (OS) in three independent cohorts used in the study: HM, GREEK, and Taber cohorts (log‐rank test, *P* = 0.038). (C) Heat map displaying molecular tumor microenvironment (TME) characteristics across all samples. The heat map includes assessments of tumor‐infiltrating immune cells estimated using CIBERSORTx, scores for different cancer ecotypes (CEs) derived from EcoTyper, and scores for consensus molecular subtypes. Additional tracks display clinical characteristics and subtypes for each patient. Overall survival (OS) is provided in months; red bars indicate events (patients who died during follow‐up), and green bars indicate censored observations (patients alive at last follow‐up). Immune cell infiltration scores and ecotype scores were column‐standardized to z‐scores for visualization in the heat map.

An overview of the analyses is shown in Fig. 1A. To investigate the impact of the immune tumor microenvironment (TME) on overall survival in patients treated with platinum‐based chemotherapy, we employed CIBERSORTx [18] in absolute mode to deconvolute the abundance of immune cell populations in each pretreatment tumor sample. CIBERSORTx utilizes gene expression signatures to characterize cellular heterogeneity from bulk tissue transcriptomic data, without the need for physical cell isolation. In the absolute mode, cellular fractions are scaled to scores that reflect each cell type's absolute proportion, allowing for comparison across both samples and cell types. As described in previous studies, the tumor‐infiltrating lymphocyte (TIL) score for each sample was determined by summing the estimated proportions of all immune cell types except eosinophils and neutrophils [19]. Monocyte derivatives were analyzed by adding estimates for macrophages (M0, M1, and M2), dendritic cells (both activated and resting dendritic cells), and monocytes. CIBERSORTx was run using the LM22 signature matrix, which characterizes 22 human immune cell subsets. Analyses were performed with B‐mode batch correction enabled, 500 permutations for statistical significance estimation, and quantile normalization disabled, as recommended for RNA‐seq input data.

We then used EcoTyper [20] to systematically identify cell states and cellular communities in the tumor samples. Cell types were first deconvolved from bulk RNA‐seq data to estimate their proportions within each sample and cell states were defined based on gene expression profiles using non‐negative matrix factorization. By analyzing the co‐occurrence patterns of these cell states across multiple samples, EcoTyper clusters cell states into recurrent multicellular communities termed “cellular ecotypes” (CEs). Each CE represents a specific configuration of cell states that reflect the functional organization of the tissue microenvironment.

Briefly, EcoTyper [20] was used to characterize 10 ecotypes. CE1‐high tumors are enriched in fibroblasts, depleted in lymphocytes, and are strongly associated with a higher risk of cancer‐associated death. CE2‐high tumors are distinguished by elevated levels of basal‐like epithelial cells, are lymphocyte‐deficient, and are associated with a significantly increased risk of mortality. CE6‐high tumors are unique because immune cell content is markedly depleted as opposed to CE9‐high tumors where proinflammatory immune cells and interferon‐gamma (IFN‐γ) activation indicate a robust immune response associated with prolonged OS. Lastly, CE10‐high tumors also have high levels of proinflammatory cells and are strongly associated with longer overall survival but are distinguished by higher B‐cell content.

Additionally, we utilized the consensus molecular classification of muscle‐invasive bladder cancer [21] implemented using the “consensusMIBC” package in R.

### 2.5 Statistical analysis

Statistical analyses included chi‐square tests for categorical variables and log‐rank tests with Cox proportional hazard regression for survival analyses. Due to potential batch effects, we analyzed CIBERSORTx results using a meta‐analysis approach. For each cohort, we performed multivariable Cox regression to evaluate associations between immune cell infiltration and overall survival, adjusted for age, T stage, and M stage. Hierarchical clustering of immune cell estimates was performed using the Euclidean distance metric and Ward's minimum variance linkage method (ward.D2). The resulting dendrogram was used to order samples for visualization purposes and was not constrained to a predetermined number of clusters.

To synthesize results across cohorts, we conducted meta‐analyses for each immune parameter. Principal component analysis of derived immune features combining CIBERSORTx immune cell fractions and ssGSEA‐based immune scores confirmed the absence of systematic batch effects in the immune‐feature estimates used for downstream analyses (Fig. S1), although gene‐level expression PCA revealed expected cohort‐driven separation, particularly for the TABER cohort, consistent with differences in RNA‐seq processing across sites. Despite this, we employed a meta‐analysis framework for several methodological advantages: (1) preservation of within‐cohort correlation structures, (2) explicit modeling of between‐study heterogeneity, (3) robustness to subtle technical variations in immune deconvolution processing, and (4) ability to weight studies by precision rather than sample size alone. This approach is particularly justified when combining retrospective cohorts with potentially different patient populations, treatment protocols, and follow‐up periods. Both fixed‐effects and random‐effects models were used to obtain pooled hazard ratios (HRs). Heterogeneity was assessed using Cochran's Q test and *I* ^2^ statistic. Meta‐analyses were conducted using the meta R package [22]. Pooled hazard ratios were calculated using the inverse‐variance weighting method. When *I* ^2^ was <50%, a common‐effect model was used for primary interpretation; when *I* ^2^ was 50% or greater, a random‐effects model based on the DerSimonian–Laird estimator was preferred.

To investigate differential impacts of cisplatin versus carboplatin, we performed subgroup analyses with patients stratified by platinum agent. Meta‐regression analyses assessed whether platinum type modified the effect of immune cell infiltration on survival outcomes. In subgroup analyses, the interaction between platinum type and immune cell infiltration was assessed using Cochran's Q test for subgroup differences. Both common‐effect and random‐effects model summaries are presented in the forest plots to facilitate comparison. The model used for primary interpretation was selected based on the *I* ^2^ heterogeneity criterion described above.

For visualizing survival‐stratification performance, we employed maximally selected rank statistics to determine optimal cutoff points that separated patients into groups with different survival outcomes. Patients were divided into high and low groups for each immune parameter, and Kaplan–Meier curves were generated. Associations between immune parameter groups and clinical/molecular variables were evaluated using appropriate statistical tests.

### 2.6 Gene expression signature analysis

To validate the potential clinical relevance of B cells in bladder cancer, we assigned four B‐cell function gene expression signatures for each tumor: (i) cytokine signaling pathways pertinent to B‐cell function; [23] (ii) B‐cell lineage gene signature; [24] (iii) lymphocyte maturation, activation, and differentiation; [25] and (iv) signature associated with B‐cell infiltration in human cancer [26]. For each signature, we calculated a score for each patient according to the methodologies described in the original publications. For signatures defined by the mean expression of the included genes, we calculated the average transcripts per million (TPM) values of these genes for each patient. For signatures with specified coefficients, we computed a weighted sum by multiplying each gene's expression level with its corresponding coefficient from the original publication.

Spearman's rank correlation coefficients were calculated between each signature score and the estimated memory B‐cell levels to determine the strength and direction of the associations. We employed the maximally selected rank statistics method to identify optimal cutoff points for stratifying patients based on their gene signature scores, identifying thresholds that most significantly differentiated patient survival outcomes without prior specification of the cutoff point. Subsequent analyses followed the same methodology as for immune‐related parameters, including survival analyses and assessment of associations with clinical and molecular variables.

To validate CIBERSORTx deconvolution results with an orthogonal approach, single‐sample gene set enrichment analysis (ssGSEA) was performed using the GSVA R package [27] (v1.50.0) with three B‐cell‐related gene sets: MCPcounter [28] B‐lineage, a custom memory B‐cell panel, and a 13‐gene TLS signature. ssGSEA normalized enrichment scores (NES) were compared with CIBERSORTx memory B‐cell fraction estimates using Spearman correlations.

To characterize the B‐cell immune niche, we integrated CIBERSORTx immune fractions, ssGSEA enrichment scores, EcoTyper ecotype assignments, and four literature‐derived B‐cell gene signatures in cisplatin‐treated patients (*n* = 167). Optimized cutoffs for memory B‐cell fraction and TLS ssGSEA NES were determined using maximally selected rank statistics, defining four immune niches: dual‐high (both above cutoff), memory‐only, TLS‐only, and dual‐low. Overall survival was compared across niches using Kaplan–Meier analysis and multivariable Cox regression adjusted for age, T stage, N stage, M stage, and MVAC treatment.

## 3 Results

### 3.1 Patient clinical characteristics

The patient and tumor characteristics are summarized in Table 1. The study group included two proprietary cohorts, the HM cohort (*n* = 55) and the GREEK cohort (*n* = 62), which were derived from a sub‐analysis of a phase III randomized clinical trial that excluded surgically eligible patients. Notably, the HM cohort included older and more medically fragile patients than the GREEK cohort did (Table 1). Across both cohorts, the most frequently administered treatment regimen was a combination of cisplatin or carboplatin and gemcitabine (*n* = 80). In the HM cohort, 22 patients received carboplatin after they were considered cisplatin‐ineligible. Additionally, we included patients with locally advanced and metastatic cancer in the external TABER cohort (*n* = 72). The TABER cohort was a publicly available clinicogenomic cohort of patients with bladder cancer from Taber et al. (Fig. 1A). The final study group consisted of 189 patients.

**Table 1:** Baseline clinical characteristics and survival outcomes of the three study cohorts. The table summarizes patient demographics, TNM staging at the time of diagnosis, treatment details, and overall survival metrics for the three study cohorts: the GREEK cohort (patients enrolled in the Hellenic Cooperative Oncology Group HE 16/03 phase III randomized trial; ACTRN12610000845033; clinical and tissue collection in Greece, 2003–2008), the HM cohort (real‐world cohort from Hospital del Mar, Barcelona, Spain; tissue and clinical data collected, 2005–2010), and the TABER cohort (publicly available clinicogenomic cohort of muscle‐invasive bladder cancer patients reported by Taber *et al*., Nat. Commun. 2020). The GREEK and TABER cohorts exclusively received cisplatin‐based chemotherapy, whereas 40% of HM cohort patients were treated with carboplatin‐based regimens. The column headed “*P* value” reports the *P* value for between‐cohort comparisons; *P* values were computed using the Kruskal–Wallis rank‐sum test for continuous variables and Pearson's χ^2^ test (or Fisher's exact test where expected counts were <5) for categorical variables. For the rows reported as percentages (T4 vs. T2/3 (%), N+ vs. N0 (%), M+ vs. M0 (%), and MVAC vs. GC (%)), the entries report the absolute number of patients in the first category followed by the corresponding within‐cohort percentage in parentheses (i.e., the number and percentage of patients in that cohort with T4 stage, node‐positive disease, metastatic disease, or MVAC‐based treatment, respectively). MVAC denotes methotrexate, vinblastine, doxorubicin, and cisplatin; GC denotes gemcitabine plus cisplatin. Significant differences were observed in age at diagnosis (*P* = 0.032) and metastatic disease at diagnosis (M+ vs. M0, *P* = 0.032). Median survival was highest in the GREEK cohort (29.8 months) and lowest in the HM cohort (17.2 months). Five‐year survival rates were poor across all cohorts, ranging from 13% (HM) to 23% (GREEK).

	GREEK cohort	HM cohort	TABER cohort	*P*‐value
No. of patients	62	55	72	
Age at diagnosis (mean (SD))	63.81 (10.7)	67.18 (8.4)	63.86 (3.3)	0.032
TNM at initial diagnosis				
T4 vs. T2/3 (%)	14 (22.6)	19 (34.5)	18 (25.0)	0.309
N+ vs. N0 (%)	28 (45.2)	26 (47.3)	40 (55.6)	0.443
M+ vs. M0 (%)	21 (33.9)	26 (47.3)	18 (25.0)	0.032
Treatment				
Any cisplatin‐based	62 (100.0)	33 (60.0)	72 (100.0)	<0.001
MVAC vs. GC (%)	35 (56.5)	3 (5.5)	2 (2.8)	<0.001
Any carboplatin‐based	0 (0.0)	22 (40.0)	0 (0.0)	<0.001
Overall survival				
Median follow‐up, months	53.6	57.6	22	
Median survival, months (95% CI)	29.8 (24.5–44.3)	17.2 (14.7–25.5)	22 (18–33)	
Fatal events (*n*)	41	42	61	
12‐month survival, % (95% CI)	89 (81–97)	70 (59–84)	75 (66–86)	
24‐month survival, % (95% CI)	65 (54–78)	34 (23–51)	47 (37–60)	
60‐month survival, % (95% CI)	23 (13–41)	13 (5.8–28)	16 (9.1–28)	

Owing to the distinct inclusion criteria across the analyzed cohorts, we observed significant differences in overall survival across the cohorts (log‐rank test, *P* = 0.038; Fig. 1B, Fig. S2A). Cohort survival differed significantly for HM versus GREEK (HR, 1.74; 95% CI: 1.13–2.68), but not the Taber cohort (HR, 1.27; 95% CI: 0.86–1.90). Patients with metastatic disease at diagnosis also had significantly shorter OS than those diagnosed with locally advanced disease (Fig. S2A–C). Moreover, patients treated with any regimen containing cisplatin showed a trend toward improved survival compared with carboplatin‐treated patients (HR 0.6; 95% CI: 0.40–1.12; *P* = 0.15; Fig. S2D). Among cisplatin‐treated patients, MVAC did not confer a statistically significant survival advantage over other cisplatin‐based regimens, although a borderline trend was observed (log‐rank test, *P* = 0.059; Fig. S2E). Finally, there was no association between survival time and age (Fig. S2F). Altogether, the 5‐year OS was poor across all cohorts, ranging from 13% to 23% (Table 1), consistent with reported outcomes for patients with advanced urothelial cancer during the study period.

### 3.2 Lymphocyte infiltration was significantly associated with prolonged overall survival

**Fig. 2:**
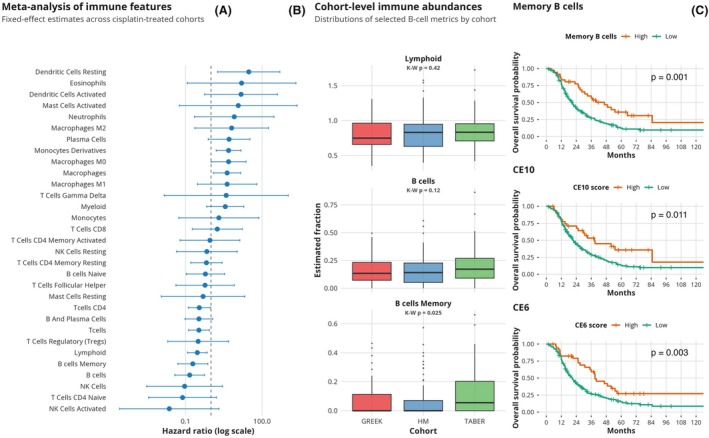
Meta‐analysis of tumor‐infiltrating immune cells. (A) Forest plots with hazard ratios (HRs) for overall survival, adjusted for age and T and M stages from the TNM (tumor‐node‐metastasis) classification. HRs for immune cell populations are estimated using CIBERSORTx. A fixed‐effects model was used when the heterogeneity statistic (*I* ^2^) was less than 50%. HR <1 indicates longer overall survival; HR >1 indicates shorter overall survival. Both common‐effect and random‐effects model estimates are presented for transparency; the primary interpretation was based on the model selected according to the *I* ^2^ criterion (common‐effect when *I* ^2^ < 50%; random‐effects otherwise; see Section 2). (B) Boxplots showing the distribution of lymphoid lineage cells, B cells, and memory B cells across the three cohorts. Box plots display median (center line), interquartile range (box), and whiskers extending to 1.5 times the interquartile range. Kruskal–Wallis testing showed no significant differences for lymphoid lineage cells (*P* = 0.42) or total B cells (*P* = 0.12) across cohorts. Memory B cells showed a statistically significant difference across cohorts (*P* = 0.025), accounted for by the random‐effects meta‐analysis framework. (C) Kaplan–Meier survival analysis for patient subgroups defined by high and low levels of memory B‐cell infiltration, CE10 and CE2 ecotype score. Subgroups were determined using the maximally selected rank statistics (“maxstat”) method (Section 2). Higher memory B‐cell infiltration (log‐rank *P* < 0.001; hazard ratio [HR] 0.47, 95% confidence interval [CI] 0.29–0.74) and CE10 scores (log‐rank *P* = 0.032) were significantly associated with improved overall survival (OS), while higher CE2 scores were associated with shorter OS (log‐rank *P* = 0.048). Subgroups were defined using the maximally selected rank statistics method.

We performed bulk RNA sequencing (RNA‐seq) for the GREEK and HM cohorts and obtained publicly available bulk RNA‐seq data for the Taber cohort. Following RNA‐seq data harmonization, clinical and transcriptomic results were projected onto a heat map (Fig. 1C). Unsupervised clustering of CIBERSORTx immune cell fractions and EcoTyper ecotype scores did not segregate samples based on cohort, molecular subtype, TNM stage, or platinum‐based regimen. However, several significant correlations were identified between immune cell infiltration levels (Fig. S3). Immune cell subpopulation analysis revealed that high levels of lymphocytes in the primary tumor were significantly associated with prolonged OS after platinum‐based chemotherapy (HR, 0.34; 95% CI: 0.16–0.72; *P* = 0.005; Fig. 2A). Notably, the association was strongest for B cells, which showed a more favorable hazard ratio than total T‐cell infiltration (HR, 0.14; 95% CI: 0.04–0.53; *P* = 0.004; Table S2; Fig. S4). Among the B‐cell subpopulations (which included naïve B cells and memory B cells), memory B cells were significantly associated with better OS (HR 0.19; 95% CI: 0.05–0.75; *P* = 0.017). The estimated levels of lymphoid and total B‐cell subgroups did not differ significantly among cohorts (Kruskal–Wallis *P* = 0.42 and *P* = 0.12, respectively), while memory B cells showed a modest but statistically significant difference across cohorts (Kruskal–Wallis *P* = 0.025; Fig. 2B), which was accounted by the random‐effects meta‐analysis framework. In contrast, the presence of myeloid cells, specifically macrophages, resting and activated dendritic cells, and neutrophils was associated with poor OS in patients with platinum‐treated urothelial cancer (Fig. S5).

To examine potential co‐regulation between B‐cell and T‐cell compartments, we assessed Spearman correlations between B‐cell and T‐cell subpopulations (Fig. S6). Memory B cells were positively correlated with activated CD4 memory T cells (ρ = 0.27, FDR‐adjusted *P* = 0.001) and resting CD4 memory T cells (ρ = 0.24, FDR‐adjusted *P* = 0.004), consistent with coordinated humoral and cellular adaptive immunity in B‐cell‐rich tumors.

To further dissect how multicellular communities correlate with OS, we applied EcoTyper [20] across tumor samples from the three cohorts. Ecotypes (CE) CE6 and CE10 were significantly associated with prolonged OS, while CE2 was associated with shorter OS, with an HR of 0.01 for CE10 (95% CI: 0.00–0.80; *P* = 0.040; Figs S7 and S14). CE10 is a proinflammatory ecosystem with high B‐cell infiltration in the tumor microenvironment (Figs S8 and S15). The ecosystem CE6 is highly specific for neoplastic tissues, which is consistent with the tumor samples profiled in this study, and CE2 is characterized by the presence of basal‐like epithelial cells associated with resistance to cisplatin chemotherapy [9].

Using cases from the three cohorts, we developed cutoffs to define tumors with high memory B‐cell infiltration (Methods). Memory B‐cell‐rich tumors comprised approximately 20% of all tumors, were evenly distributed across cohorts (chi‐squared test; *P* = 0.177), and were associated with a significantly better prognosis (Fig. 2C; HR 0.47; 95% CI: 0.29–0.74). Moreover, high memory B‐cell presence was associated with CE10 (chi‐squared test; *P* < 0.001) and CE6 (chi‐squared test; *P* = 0.003) ecosystems, consistent with the EcoTyper‐expected immune cell infiltration.

Together, these results indicate that the presence of memory B cells and a B‐cell‐rich ecosystem in primary muscle‐invasive bladder tumors is associated with improved OS in patients with advanced muscle‐invasive bladder cancer treated with systemic platinum‐based chemotherapy.

### 3.3 Proinflammatory tumor microenvironment is associated with prolonged overall survival following cisplatin treatment

**Fig. 3:**
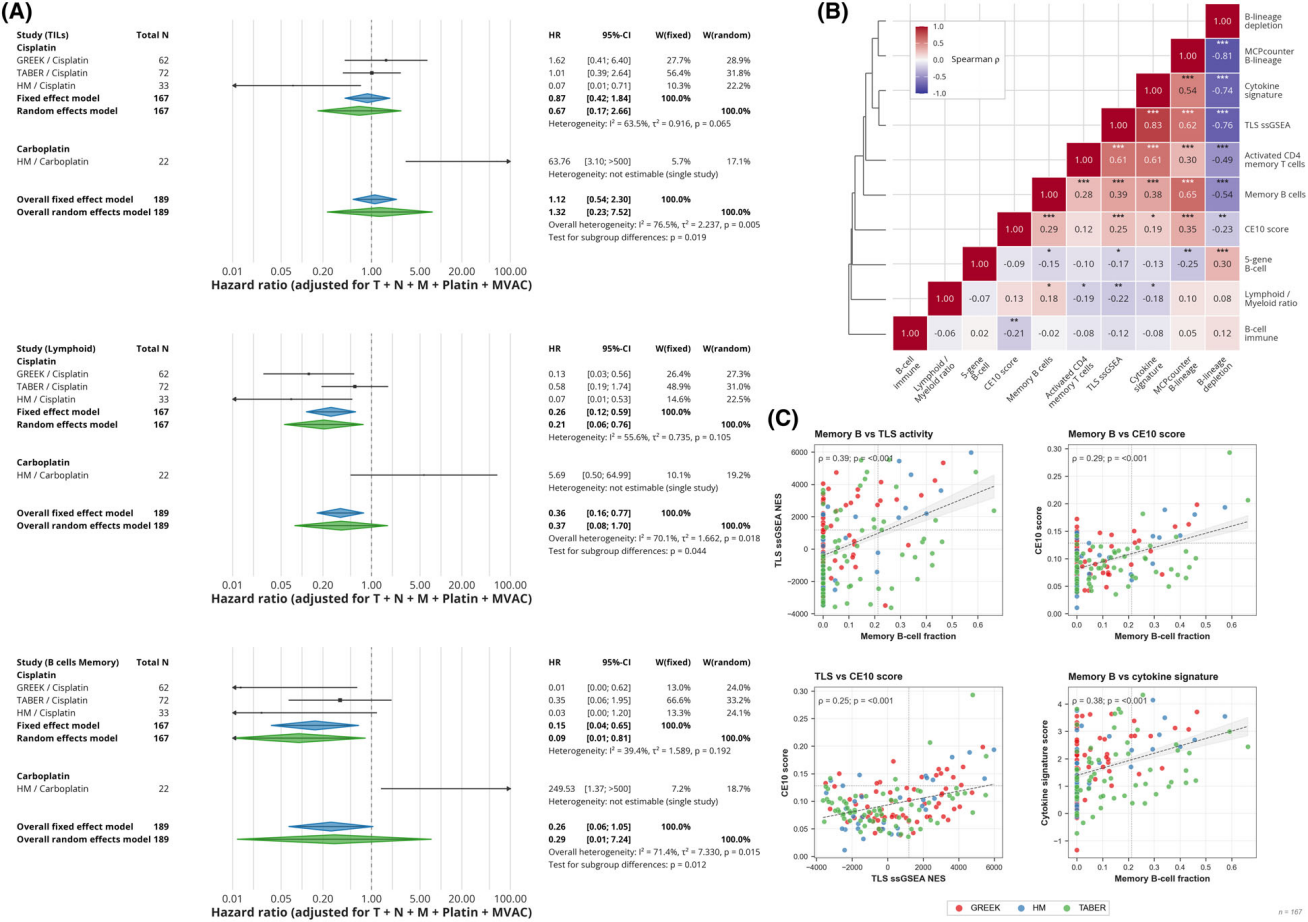
Integrative figure linking platinum‐treatment meta‐analysis, B‐cell immune‐score correlations, and scatter relationships in cisplatin‐treated tumors. (A) Meta‐analysis forest results comparing cisplatin‐ and carboplatin‐treated groups for lymphoid metrics; subgroup difference P values for total tumor‐infiltrating lymphocytes (TILs; *P* = 0.019), lymphoid lineage cells (*P* = 0.044), and memory B cells (*P* = 0.012) are reported in the panel. Whiskers indicate 95% confidence intervals (95% CI) of cohort‐level hazard ratios (HRs); diamonds indicate the pooled HR. (B) Correlation heat map between B‐cell‐related immune scores/fractions and linked ecotype or single‐sample gene set enrichment analysis (ssGSEA) measures; color intensity reflects the magnitude of the Spearman rank correlation coefficient. (C) Scatter plots showing pairwise associations, including memory B‐cell fraction versus the ssGSEA normalized enrichment score (NES) for the 13‐gene tertiary lymphoid structure (TLS) signature (TLS ssGSEA NES). Spearman rank correlation coefficients (ρ) and corresponding P values are displayed on each scatter plot; key associations include memory B‐cell fraction versus TLS ssGSEA NES (ρ = 0.39, *P* < 0.001), CD4 T cells versus TLS ssGSEA NES (ρ = 0.59, *P* < 0.001), and CD8 T cells versus TLS ssGSEA NES (ρ = 0.23, *P* = 0.003). The shaded gray area in each scatter plot represents the 95% CI of the linear regression line. CE, cellular ecotype; CI, confidence interval; HR, hazard ratio; NES, normalized enrichment score; OS, overall survival; ssGSEA, single‐sample gene set enrichment analysis; TILs, tumor‐infiltrating lymphocytes; TLS, tertiary lymphoid structures. Forest plots display hazard ratios (HRs) for overall survival associated with tumor‐infiltrating lymphocytes (TILs) (A), lymphoid lineage cells (B), and memory B cells (C), adjusted for age and T and M stage. A fixed‐effects model was used when the heterogeneity statistic (*I* ^2^) was <50%. Significant differences in HRs between the cisplatin and carboplatin groups were observed, confirmed by tests for subgroup differences (interaction *P*‐values), indicating that immune cell infiltration was associated with improved overall survival in cisplatin‐treated patients, but not in those treated with carboplatin (TILs subgroup difference *P* = 0.019; lymphoid lineage subgroup difference *P* = 0.044; memory B‐cell subgroup difference *P* = 0.012). Correlation analyses (panel B) used Spearman rank correlation. Scatter plots (panel C) show Spearman rank correlations with 95% confidence intervals (shaded gray area). CI, confidence interval; HR, hazard ratio; OS, overall survival; TILs, tumor‐infiltrating lymphocytes. HR <1 indicates longer overall survival; HR >1 indicates shorter overall survival. Both common‐effect and random‐effects model estimates are shown; the model used for primary interpretation was selected based on the *I* ^2^ heterogeneity criterion (see Section 2).

To investigate whether cisplatin and carboplatin differentially modulate the bladder tumor microenvironment and the impact of these changes on OS, we divided the cohorts based on the treatment regimen (Table S3). Of the 189 patients included in the study, 167 received cisplatin‐based chemotherapy, and 22 received carboplatin‐based chemotherapy. Tumor‐infiltrating lymphocytes (TILs) showed a significant differential association with OS by platinum type (subgroup difference, *P* = 0.019; Fig. 3A), with a trend toward better OS in cisplatin‐treated patients that did not reach statistical significance (HR 0.87, *P* = 0.72). Among specific cell types, lymphoid lineage cells showed the strongest differential association favoring cisplatin‐treated patients (subgroup difference, *P* = 0.044; Fig. 3A), particularly memory B cells (subgroup difference, *P* = 0.012; Fig. 3A; Fig. S9, within‐carboplatin *P* = 0.037). We then defined differences in ecosystems in patients treated with cisplatin versus carboplatin and found that CE10 (proinflammatory and B‐cell‐rich) ecosystems showed a trend toward favorable association in cisplatin‐treated patients, though the subgroup difference did not reach statistical significance (common effect model, HR 0.01; 95% CI: 0.00–1.62). CE10 score distributions by platinum type are shown in Fig. S10A. Additionally, intergroup differences were noted for neutrophils and the LumNS consensus subtype, in line with the fact that LumNS tumors displayed elevated stromal infiltration signatures, primarily fibroblastic, compared with other luminal tumors (Fig. S10B). Overall, these results indicate that immune cell infiltration, particularly of memory B cells and the proinflammatory CE10 ecotype, is associated with prolonged OS following cisplatin but not carboplatin chemotherapy.

### 3.4 Integrative characterization of the memory B‐cell/TLS immune niche

**Fig. 4:**
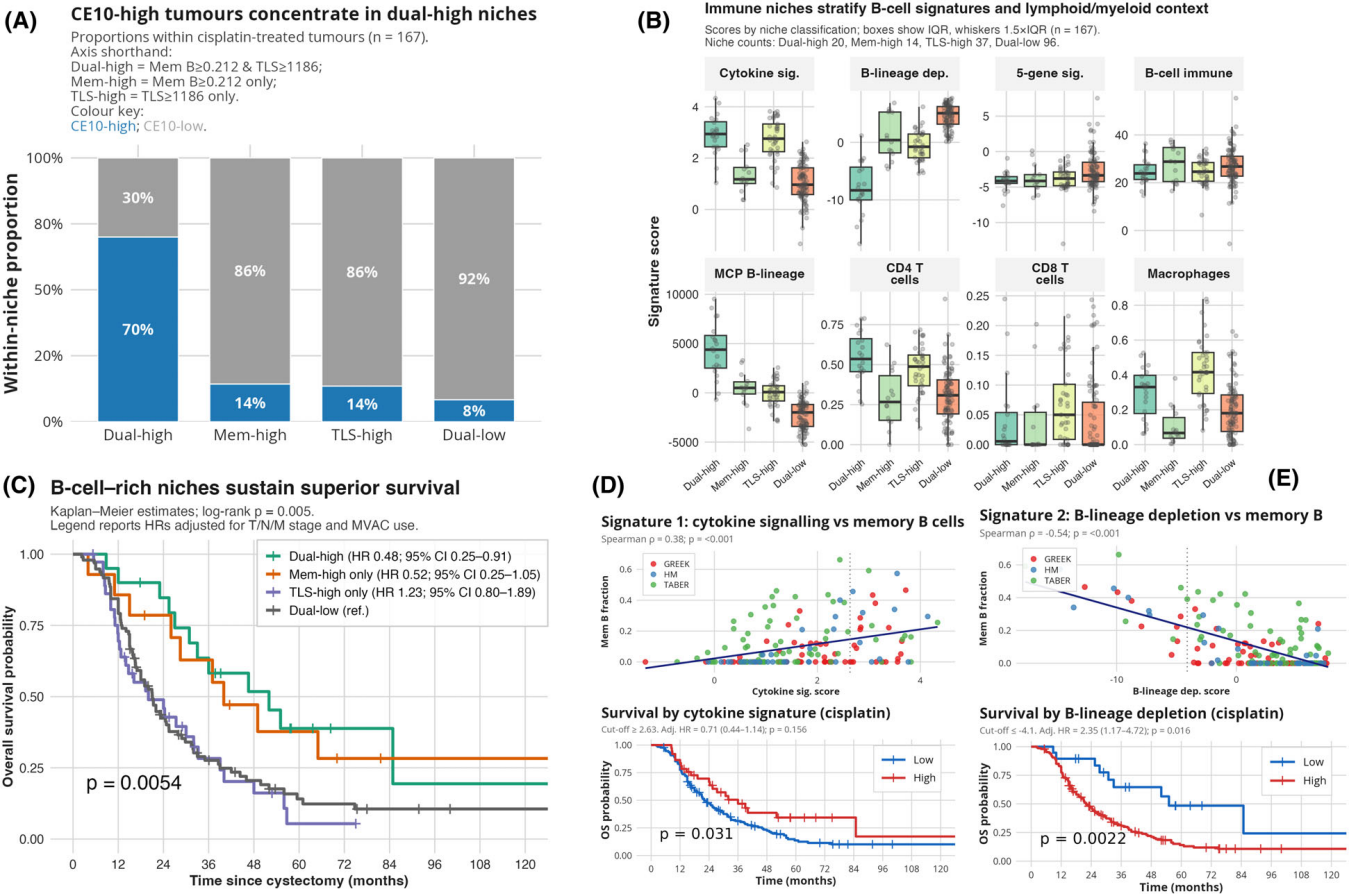
Integrative niche analysis and B‐cell signature validation in cisplatin‐treated bladder cancer. (A) CE10‐high prevalence across four immune niches (dual‐high, memory‐high, TLS‐high, and dual‐low). (B) Immune‐niche stratification of B‐cell signatures, MCPcounter B‐lineage, and CIBERSORTx CD4/CD8 T‐cell and macrophage fractions. (C) Kaplan–Meier survival by immune niche in the cisplatin‐treated cohort. (D) Cytokine signaling signature score versus memory B‐cell fraction with Kaplan–Meier survival stratification. (E) B‐cell lineage depletion signature score versus memory B‐cell fraction with Kaplan–Meier survival stratification. CE, cellular ecotype; CI, confidence interval; HR, hazard ratio; NES, normalized enrichment score; OS, overall survival; TLS, tertiary lymphoid structures. Box plots (panel B) display the median (center line), the interquartile range (box: 25th–75th percentiles), and whiskers extending to 1.5 times the interquartile range; individual data points are overlaid. Thresholds: memory B‐cell fraction ≥ 0.212; TLS ssGSEA NES ≥ 1186; CE10 score ≥ 0.128. All survival analyses are restricted to cisplatin‐treated patients (*n* = 167). Statistical tests: Kruskal–Wallis (B), Spearman rank correlations (D, E), and log‐rank/Cox proportional hazards models (C–E). Adjusted Cox models include T stage, N stage, M stage, and treatment with MVAC (methotrexate, vinblastine, doxorubicin, and cisplatin) as covariates.

Correlation structure among immune fractions and B‐cell signatures is shown in Fig. 3B, and scatter analyses including memory B versus TLS are shown in Fig. 3C. To further characterize the favorable immune microenvironment associated with memory B cells, we performed an integrative analysis combining CIBERSORTx immune fractions, ssGSEA enrichment scores, and EcoTyper ecotype assignments in cisplatin‐treated patients (*n* = 167; Fig. 4). Using optimized cut‐offs derived from maximally selected rank statistics, we defined four immune niches: dual‐high (high memory B cells and high TLS activity), memory‐high, TLS‐high, and dual‐low. CE10‐high status is concentrated in the dual‐high niche (Fig. 4A). Immune‐niche stratification boxplots are shown in Fig. 4B,C compares survival by niche. In multivariable analysis adjusted for age, T stage, N stage, M stage, and MVAC treatment, high memory B‐cell infiltration was independently associated with improved overall survival (HR 0.48; 95% CI: 0.29–0.80; *P* = 0.005), and CE10‐high status was similarly associated with improved overall survival (HR 0.51; 95% CI: 0.30–0.87; *P* = 0.013). Kaplan–Meier analysis confirmed that the dual‐high niche showed the most favorable survival pattern, although the niche‐level comparison was marginally significant (dual‐high vs. dual‐low HR 0.54; 95% CI: 0.29–1.01; *P* = 0.055). To validate these CIBERSORTx‐derived findings with an orthogonal method, we performed ssGSEA using three gene sets. TLS ssGSEA enrichment showed concordance with CIBERSORTx memory B‐cell fractions (ρ = 0.39, *P* < 0.001) but not total B cells (ρ = 0.02, *P* = 0.803), and moderate‐to‐strong correlations with T‐cell estimates (CD4 T cells ρ = 0.59, *P* < 0.001; CD8 T cells ρ = 0.23, *P* = 0.003; Fig. S16), and ssGSEA scores correlated strongly with CIBERSORTx memory B‐cell estimates (MCPcounter B‐lineage ρ = 0.64, *P* < 0.001; Figs S16, S17). In multivariable Cox models, higher MCPcounter B‐lineage ssGSEA scores were associated with improved OS (adjusted HR = 0.91; 95% CI: 0.85–0.99; *P* = 0.019; Fig. S18).

Four B‐cell gene expression signatures previously validated in other cancer types were evaluated for concordance with memory B‐cell infiltration and survival stratification performance (Fig. 4D,E; Fig. S11). A cytokine signaling signature [23] correlated positively with memory B‐cell infiltration (ρ = 0.38, *P* < 0.001; Fig. 4D) and was associated with improved OS in univariate analysis of cisplatin‐treated patients (HR = 0.61, 95% CI: 0.38–0.96, *P* = 0.032; log‐rank *P* = 0.031) but did not retain independent significance after covariate adjustment (adjusted HR = 0.71, 95% CI: 0.44–1.14, *P* = 0.156; covariates: T/N/M stage, MVAC). A B‐cell lineage depletion signature [24] showed an inverse correlation with memory B cells (ρ = −0.54, *P* < 0.001; Fig. 4E) and was independently associated with worse OS after covariate adjustment (adjusted HR = 2.35, 95% CI: 1.17–4.72, *P* = 0.016; covariates: T/N/M stage, MVAC; log‐rank *P* = 0.002), with a significant platinum‐type interaction (subgroup *P* = 0.005; Fig. S11). The remaining two signatures (5‐gene B‐cell [26] and 14‐gene B‐cell immune [25]) did not reach statistical significance for correlation with memory B cells or survival stratification in the cisplatin subgroup. Overall, these results demonstrate that memory B‐cell infiltration, TLS activity, and the CE10 ecotype form a coherent immune niche associated with favorable outcomes in cisplatin‐treated patients, validated by multiple orthogonal approaches.

## 4 Discussion

In this study, we found that lymphocyte infiltration, particularly memory B cells, was associated with prolonged overall survival in patients receiving cisplatin‐based chemotherapy for advanced muscle‐invasive urothelial carcinoma. Notably, this association was not observed in patients treated with carboplatin‐based chemotherapy. Taken together, these data suggest a treatment context‐dependent, predictive association of memory B‐cell‐rich tumors in this cohort, rather than a direct causal treatment effect.

These results suggest differential treatment‐context associations between cisplatin and carboplatin related to immune activation, which should be interpreted as hypothesis‐generating in this retrospective setting. Cisplatin induces immunogenic cell death, leading to tumor antigen release and activation of dendritic cells and T cells [29]. These observations are consistent with this framework, as selected markers of T‐cell activation (CD69, *P* < 0.001) and dendritic cell activation (CCR7, *P* < 0.001) were significantly upregulated in specimens with high memory B‐cell infiltration, with trends toward an increased expression of additional markers including IFNG, IL2RA, and CD86 (Fig. S12). A recent subanalysis of IMvigor130 [5] showed that cisplatin, but not carboplatin, exerts direct immunomodulatory effects on cancer cells, promoting dendritic cell activation and antigen‐specific T‐cell killing, consistent with our observations.

It should be noted that the platinum agents in our study were predominantly administered in combination with gemcitabine, which has known immunomodulatory properties including depletion of myeloid‐derived suppressor cells and enhancement of cross‐priming of tumor‐specific T cells. These gemcitabine‐mediated effects may independently influence the observed immune profiles, and the relative contributions of the platinum agent versus gemcitabine cannot be fully disentangled in the current analysis. Furthermore, a subset of patients in the GREEK cohort received DD‐MVAC rather than gemcitabine–platinum combinations, introducing additional heterogeneity in chemotherapy backbones across cohorts.

The identification of specific ecosystems, particularly CE10 (pro‐inflammatory and B cell‐rich), provides additional evidence for the clinical relevance of immune cell populations. Our integrative niche analysis (Fig. 4) demonstrated that tumors with both high memory B‐cell infiltration and high TLS activity constitute a distinct immune niche with favorable outcomes, reinforcing the biological coherence of the memory B‐cell/TLS/CE10 axis. Ecotype CE10 is becoming established as a predictive TME factor associated with immunotherapy response across cancer types [30]. Our results demonstrate that B‐cell‐rich environments strongly correlate with improved cisplatin efficacy, suggesting both predictive utility and potential therapeutic strategies aimed at enhancing B‐cell recruitment and tertiary lymphoid structure formation to improve treatment outcomes.

The modulation of B‐cell infiltration represents a promising approach for enhancing antitumor immunity, as effective cooperation between B cells and T cells within the tumor microenvironment may lead to regression of established tumors [31]. Targeting the CXCL13‐CXCR5 axis presents a compelling strategy, as CXCL13 mediates recruitment of B cells to tumors and is essential for the formation of tertiary lymphoid structures that support antitumor immunity [21]. Immune cell infiltration can also be modulated by radiotherapy [32].

Future studies should incorporate spatial profiling approaches to further characterize the B‐cell niche in platinum‐treated muscle‐invasive bladder tumors. Spatial transcriptomics or multiplexed immunohistochemistry could localize memory B cells relative to tertiary lymphoid structures (TLS), and assessment of TLS density and maturation from H&E‐stained sections would provide additional insight into immune organization. Immunofluorescence‐based co‐localization of memory B‐cell markers with TLS components would further elucidate whether memory B cells are primarily TLS‐associated or also present as dispersed populations within the tumor parenchyma, providing functional context for the transcriptomic signatures identified in the current study.

Interestingly, we did not observe significant associations between prognosis, immune cell infiltration, and consensus molecular clustering. This finding aligns with the known heterogeneity within molecular subtypes, where immune infiltration patterns can vary substantially within the same subtype classification. Mixed validation results continue to hinder the clinical translation of molecular subtypes [33], suggesting that transcriptomic classifiers may not fully capture the immune contexture of individual tumors. The consistency of our findings across the cytokine signaling and B‐cell lineage depletion signatures—originally developed and validated in other cancer types including lung, pancreatic, and breast cancer—suggests that B‐cell‐related gene expression signatures capture conserved biological programs of B‐cell infiltration that transcend individual tumor types. These cross‐cancer validated signatures may therefore provide more robust survival stratification information than molecular subtype classifiers for identifying patients with B‐cell‐rich tumor microenvironments. Notably, the 5‐gene B‐cell signature derived from pancreatic adenocarcinoma showed an anomalous negative correlation with other B‐cell signatures and a positive correlation with the B‐lineage depletion signature (Fig. 3B), suggesting that this particular signature may capture a distinct biological program not reflective of canonical B‐cell infiltration in urothelial cancer.

This study has several limitations. The retrospective design introduces potential selection bias and limits causal inferences. Although we adjusted for confounding variables such as age and tumor stage, unmeasured factors like performance status and comorbidities could influence outcomes. Heterogeneity among the three cohorts and the small carboplatin‐treated sample (*n* = 22) may affect generalizability and statistical power. Additionally, computational estimations of immune cell populations from bulk RNA‐seq may not capture the full diversity of the tumor microenvironment, and potential batch effects were mitigated but not eliminated. Stage‐stratified analysis revealed that the favorable association of memory B‐cell infiltration with overall survival was consistent in direction across locally advanced (HR = 0.53, *P* = 0.056) and metastatic (HR = 0.55, *P* = 0.097) subgroups (Fig. S13), though individual strata lacked statistical power due to reduced sample sizes. Significant age differences existed between cohorts (*P* = 0.032), with the HM cohort—the only cohort including carboplatin‐treated patients—having older patients. Age‐related immunosenescence may independently affect immune cell infiltration estimates and confound comparisons between platinum agents.

Carboplatin is typically administered to patients deemed cisplatin‐ineligible due to impaired renal function, poor performance status, or significant comorbidities. This inherent selection bias means that carboplatin‐treated patients may have pre‐existing impairment of immune function, potentially confounding the observed differences in immune cell infiltration between treatment groups independently of any direct pharmacological effect.

Furthermore, all carboplatin‐treated patients (*n* = 22) originated from a single cohort (HM), precluding independent validation of carboplatin‐specific findings across cohorts. The extremely small subgroup sizes in immune‐enriched carboplatin‐treated patients (2–3 patients in some strata; Fig. S9) are insufficient to draw reliable conclusions about the interaction between immune enrichment and carboplatin treatment. Accordingly, this study is underpowered for carboplatin‐specific conclusions, and all findings related to differential effects of carboplatin should be interpreted with appropriate caution.

Additionally, the optimal thresholds used to stratify patients into high and low immune infiltration groups were derived from the same meta‐dataset without subsequent validation in an independent or held‐out cohort. This data‐dependent threshold selection may lead to overestimation of survival stratification performance, and external validation in independent cohorts is needed to confirm the robustness of these cutoffs.

## 5 Conclusions

Our study demonstrates a significant association between memory B‐cell infiltration and prolonged overall survival in advanced muscle‐invasive urothelial bladder cancer patients treated with cisplatin‐based chemotherapy. The observed differences across platinum groups and ecosystem patterns are hypothesis‐generating and should be considered in the context of retrospective design and cohort heterogeneity. Because our analysis is observational, causal claims cannot be made. Importantly, these findings provide a rationale for prospective studies testing whether interventions that modulate B‐cell infiltration or tertiary lymphoid structure formation can improve outcomes in this treatment setting.

## Conflict of interest

JB reports the following financial interests: advisory board participation with AstraZeneca, BMS, Merck, and Pfizer; participation as an invited speaker or lecturer by Merck and MSD; stocks and/or shares from Bicycle; royalties from UpToDate. The authors declare that they have no affiliations with or involvement in any organization or entity with any financial interest in the subject matter or materials discussed in this manuscript.

## Author contributions

All authors meet the four authorship criteria of the International Committee of Medical Journal Editors (ICMJE): (1) substantial contributions to the conception or design of the work, or to the acquisition, analysis, or interpretation of data; (2) drafting the work or revising it critically for important intellectual content; (3) final approval of the version to be published; and (4) agreement to be accountable for all aspects of the work, ensuring that questions related to the accuracy or integrity of any part of the work are appropriately investigated and resolved. Specific contributions are as follows. KS and JPB contributed equally to this work. KS, JPB, FLFC, and JB conceived and designed the study. ARV, NJ, and AB acquired clinical samples and patient/follow‐up data; NJ performed pathological review and sample selection; AB led patient enrollment and data acquisition for the GREEK cohort; ARV led data acquisition for the HM cohort. JL and DEM performed RNA sequencing and primary data processing. KS and JPB performed the bioinformatic and statistical analyses and interpreted the data, with critical intellectual input from JLG, KWM, FLFC, and JB on the immune analyses and clinical interpretation. KS and JPB drafted the original manuscript. ARV, NJ, JL, DEM, JLG, KWM, AB, FLFC, and JB revised the manuscript critically for important intellectual content. FLFC, KWM, and KS acquired funding. FLFC and JB supervised the project and provided resources. All authors reviewed and approved the final version of the manuscript and agree to be accountable for all aspects of the work.

## Supporting information


**Fig. S1.** Principal component analysis (PCA) of (A) gene‐level expression data and (B) derived immune features combining CIBERSORTx immune cell fractions and ssGSEA‐based immune scores, colored by cohort.
**Fig. S2.** Univariable overall survival analysis of clinical and treatment covariates.
**Fig. S3.** Network plot of significant correlations between molecular metrics.
**Fig. S4.** Forest plot of Cox proportional hazards models adjusted for clinical covariates, evaluating total T‐cell infiltration and overall survival in each cohort with a pooled estimate.
**Fig. S5.** Forest plots of meta‐analysis evaluating the association between myeloid cell infiltration and overall survival.
**Fig. S6.** Spearman correlations between B‐cell and T‐cell subpopulations estimated by CIBERSORTx (*n* = 189).
**Fig. S7.** Kaplan–Meier analysis of overall survival for different ecotypes.
**Fig. S8.** Correlations between B‐cell memory infiltration and CE10, CE7, and CE6 scores.
**Fig. S9.** Kaplan–Meier analysis within carboplatin‐treated patients.
**Fig. S10.** Differences in immune cell infiltration and molecular subtype scores between cisplatin‐ and carboplatin‐treated patients.
**Fig. S11.** Association of B‐cell‐related gene signatures with overall survival in cisplatin‐ versus carboplatin‐treated patients.
**Fig. S12.** Differential expression of immune activation markers in tumors with high versus low memory B‐cell infiltration.
**Fig. S13.** Stage‐stratified analysis of memory B‐cell infiltration and overall survival in cisplatin‐treated patients.
**Fig. S14.** Association between tumor ecotypes and overall survival.
**Fig. S15.** Comparison of immune cell infiltration levels in tumors with high versus low memory B‐cell infiltration.
**Fig. S16.** Validation of CIBERSORTx memory B‐cell fraction estimates using single‐sample gene set enrichment analysis (ssGSEA) enrichment scores.
**Fig. S17.** Summary of Spearman rank correlations between CIBERSORTx memory B‐cell fractions and ssGSEA normalized enrichment scores for the MCPcounter B‐lineage, custom memory B‐cell, and 13‐gene TLS signatures.
**Fig. S18.** Adjusted Cox proportional hazards models for ssGSEA‐derived signature scores and overall survival.

**Table S1.** Clinical characteristics of included patients. TNM stage at the time of diagnosis.
**Table S2.** Meta‐analysis results for all established parameters.
**Table S3.** Meta‐analysis results for all established parameters with assessment of differences between cisplatin‐ and carboplatin‐treated patients.

## Data Availability

The code and files used to generate all figures are available at https://github.com/CarvalhoFilipeL/bladder_cancer_B_cell_sigs. Raw RNA‐seq reads were deposited in GEO at the time of publication.
