# Supplementary material for: Tumor B‐cell infiltration in platinum‐treated advanced muscle‐invasive urothelial carcinoma
Source: Mol Oncol. 2026 Jun 1:10.1002/1878-0261.70276. Online ahead of print. doi: 10.1002/1878-0261.70276 (PMC13398980; doi:10.1002/1878-0261.70276)
Supplement: Supplementary file 1 — Fig. S1. Principal component analysis (PCA) of (A) gene‐level expression data and (B) derived immune features combining CIBERSORTx immune cell fractions and ssGSEA‐based immune scores, colored by cohort. Fig. S2. Univariable overall survival analysis of clinical and treatment covariates. Fig. S3. Network plot of significant correlations between molecular metrics. Fig. S4. Forest plot of Cox proportional hazards models adjusted for clinical covariates, evaluating total T‐cell infiltration and overall survival in each cohort with a pooled estimate. Fig. S5. Forest plots of meta‐analysis evaluating the association between myeloid cell infiltration and overall survival. Fig. S6. Spearman correlations between B‐cell and T‐cell subpopulations estimated by CIBERSORTx (n = 189). Fig. S7. Kaplan–Meier analysis of overall survival for different ecotypes. Fig. S8. Correlations between B‐cell memory infiltration and CE10, CE7, and CE6 scores. Fig. S9. Kaplan–Meier analysis within carboplatin‐treated patients. Fig. S10. Differences in immune cell infiltration and molecular subtype scores between cisplatin‐ and carboplatin‐treated patients. Fig. S11. Association of B‐cell‐related gene signatures with overall survival in cisplatin‐ versus carboplatin‐treated patients. Fig. S12. Differential expression of immune activation markers in tumors with high versus low memory B‐cell infiltration. Fig. S13. Stage‐stratified analysis of memory B‐cell infiltration and overall survival in cisplatin‐treated patients. Fig. S14. Association between tumor ecotypes and overall survival. Fig. S15. Comparison of immune cell infiltration levels in tumors with high versus low memory B‐cell infiltration. Fig. S16. Validation of CIBERSORTx memory B‐cell fraction estimates using single‐sample gene set enrichment analysis (ssGSEA) enrichment scores. Fig. S17. Summary of Spearman rank correlations between CIBERSORTx memory B‐cell fractions and ssGSEA normalized enrichment scores for the MCPcounter B‐lineage [file MOL2-9999-0-s002.pdf]

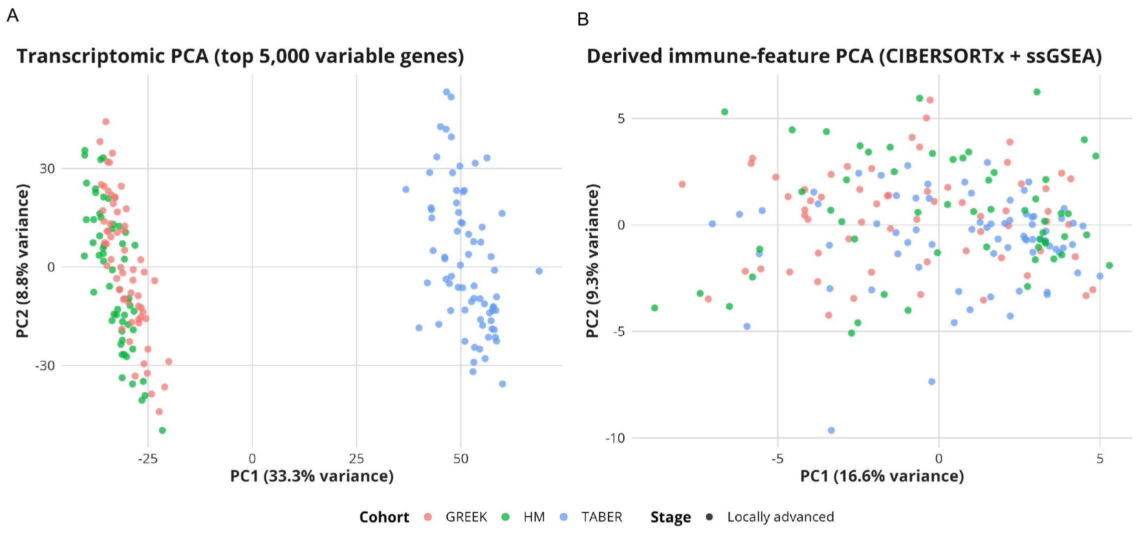

Supplementary Figure 1. Principal component analysis (PCA) of (A) gene-level expression data and (B) derived immune features combining CIBERSORTx immune cell fractions and ssGSEA-based immune scores, colored by cohort. Gene-level PCA reveals expected cohort-driven separation, particularly for the TABER cohort, while the derived immune-feature PCA shows no systematic batch effects, supporting the use of immune-feature estimates for cross-cohort meta-analysis.

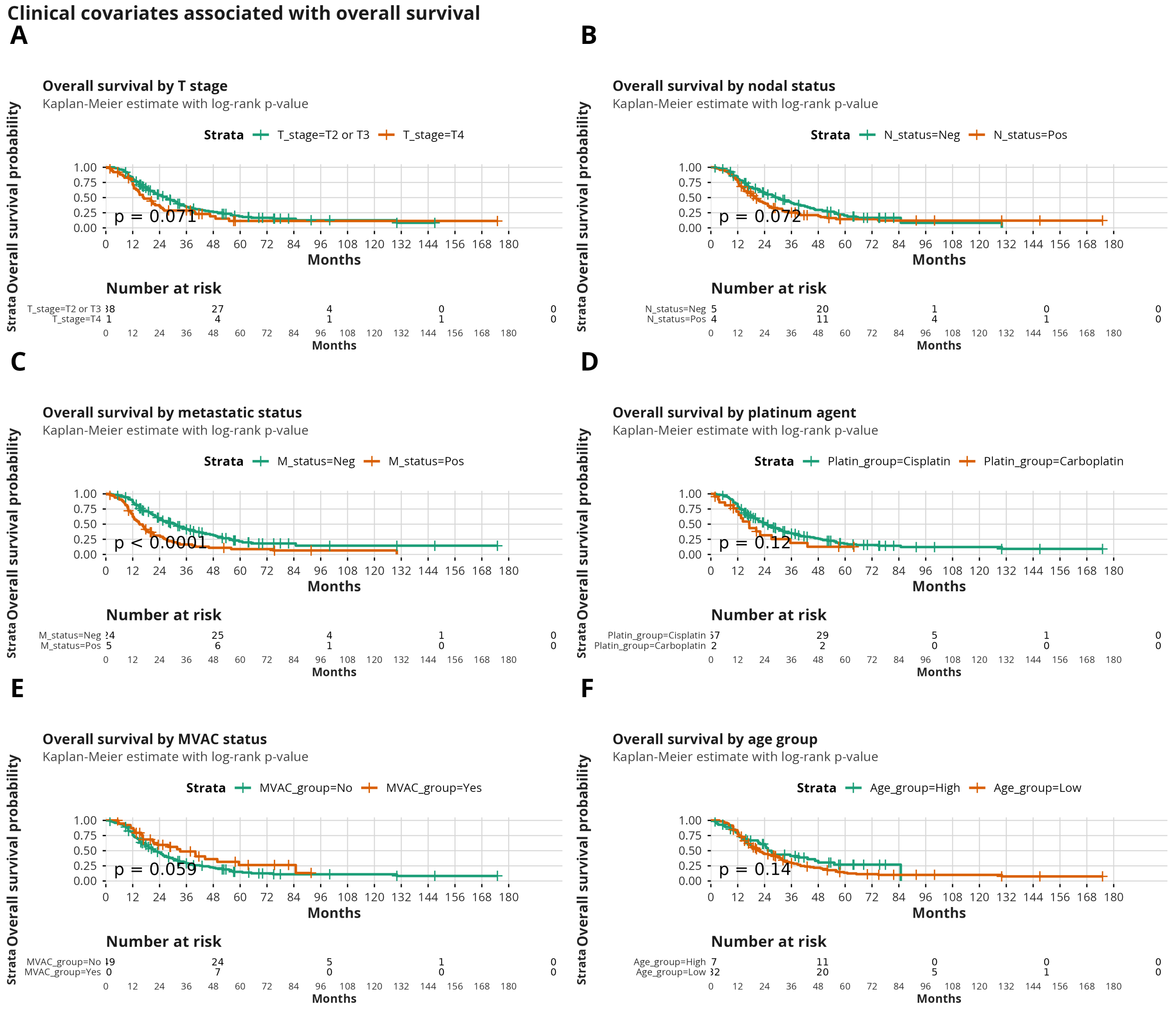

Supplementary Figure 2. Univariable overall survival analysis of clinical and treatment covariates. Kaplan–Meier estimates of overall survival across the pooled cohort. (A) Overall survival by T stage (T2/T3 versus T4). (B) Overall survival by nodal status (N0 versus N+). (C) Overall survival by metastatic status (M0 versus M+). (D) Overall survival by platinum agent (cisplatin versus carboplatin). (E) Overall survival by MVAC versus non-MVAC chemotherapy regimen. (F) Overall survival by age group (high versus low; cut-off optimized using maximally selected rank statistics). P values shown on each plot were calculated using the log-rank test. The risk table beneath each Kaplan–Meier curve reports the number of patients at risk in each stratum. TNM = tumor-node-metastasis staging system; MVAC = methotrexate, vinblastine, doxorubicin, and cisplatin; OS = overall survival.

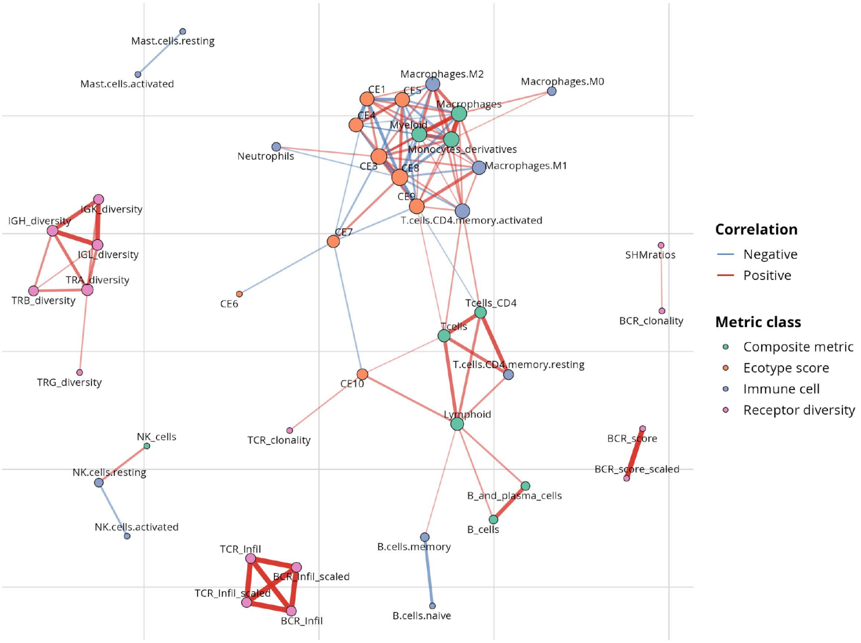

Supplementary Figure 3. Network plot of significant correlations between molecular metrics. This network visualizes the significant correlations between parameters (correlation coefficient |r| > 0.4, p < 0.05). Nodes represent individual immune cell populations or molecular metrics, while edges represent significant positive (red) or negative (blue) correlations between them. The thickness of the edges corresponds to the strength of the correlation, with thicker lines indicating stronger associations.

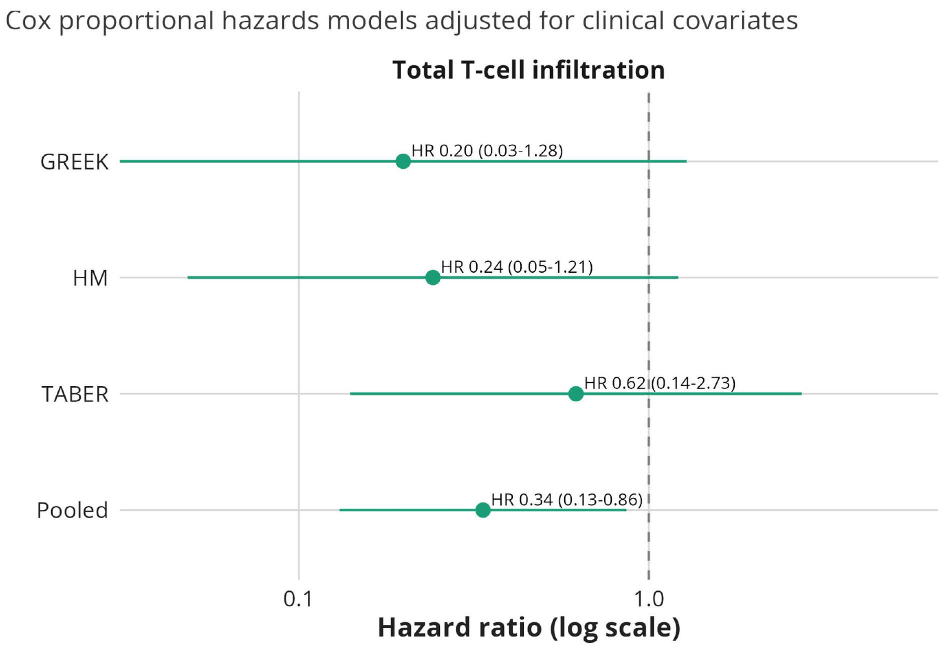

Supplementary Figure 4. Forest plot of Cox proportional hazards models adjusted for clinical covariates, evaluating total T-cell infiltration and overall survival in each cohort with a pooled estimate. Higher total T-cell infiltration was associated with improved survival in the pooled analysis (HR = 0.34; 95% CI: 0.13–0.86), although the association was less pronounced than that observed for B-cell infiltration.

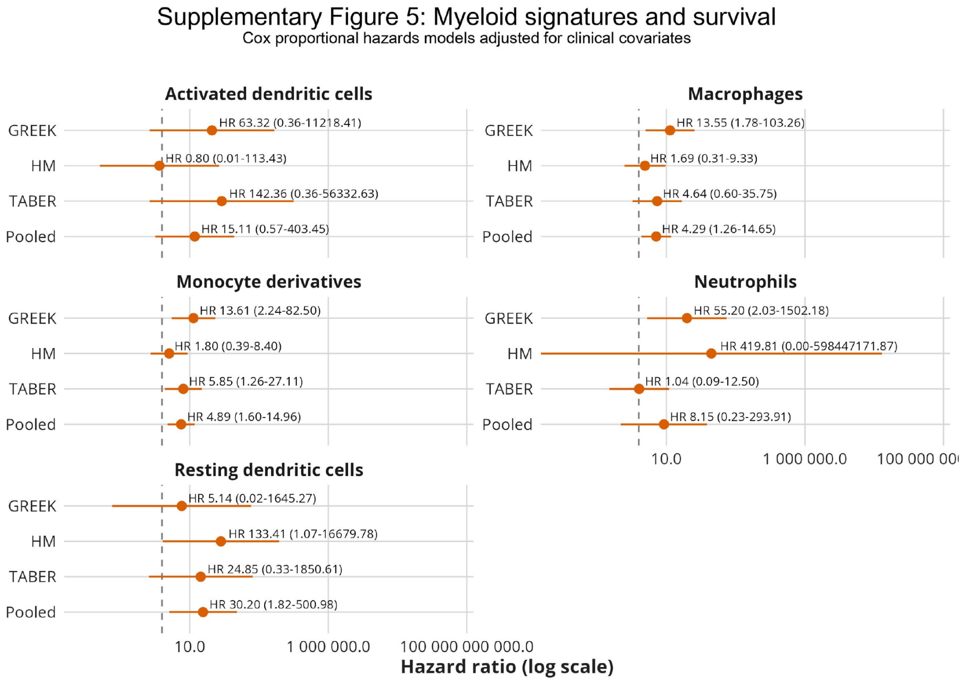

Supplementary Figure 5. Forest plots of meta-analysis evaluating the association between myeloid cell infiltration and overall survival. The figure displays hazard ratios (HRs) and 95% confidence intervals (CIs) for monocytes derivatives and neutrophils in each cohort and the pooled estimates, indicating an association with poorer survival.

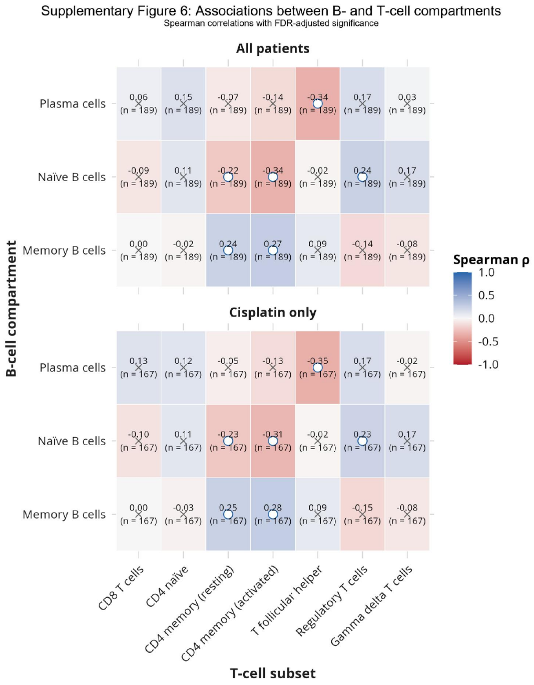

Supplementary Figure 6. Spearman correlations between B-cell and T-cell subpopulations estimated by CIBERSORTx (n = 189). Memory B cells showed significant positive correlations with activated CD4 memory T cells (ρ = 0.27, FDR-adjusted p = 0.001) and resting CD4 memory T cells (ρ = 0.24, FDR-adjusted p = 0.004).

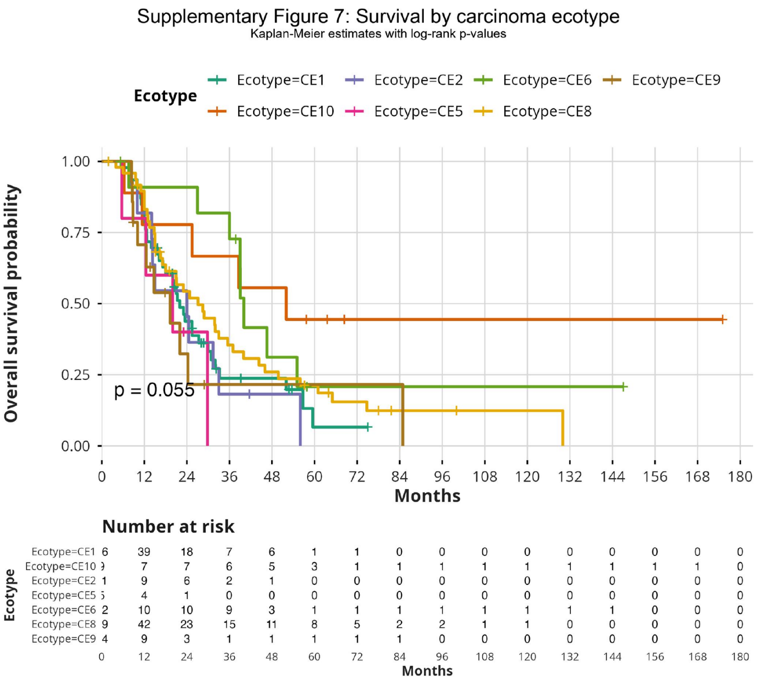

Supplementary Figure 7. Kaplan-Meier analysis of overall survival for different Ecotypes. Log-rank test showed borderline significance for differences between ecotypes (log-rank p = 0.068).

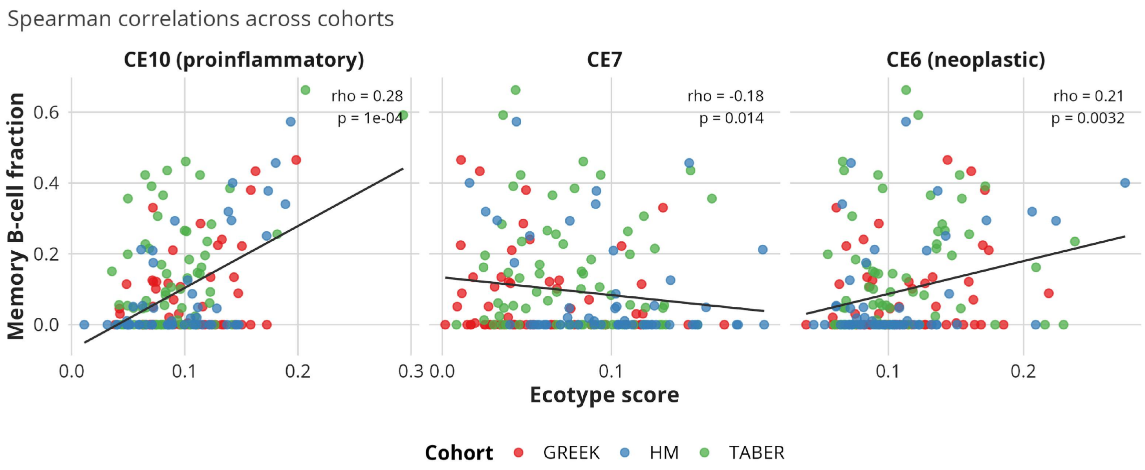

Supplementary Figure 8. Correlations between B cell memory infiltration and CE10, CE7, and CE6 scores. Spearman rank correlation coefficients and p-values are shown. Memory B-cell fraction showed a significant positive correlation with CE10 score and no significant correlation with CE7 or CE6 scores.

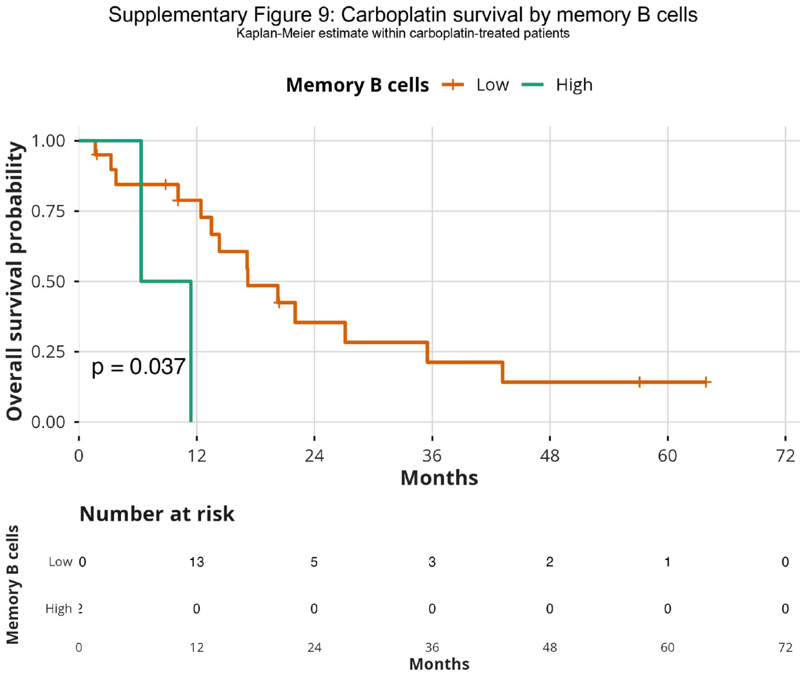

Supplementary Figure 9. Kaplan–Meier analysis within carboplatin-treated patients. Higher memory B-cell infiltration was associated with worse overall survival within the carboplatin subgroup (log-rank p = 0.037), but this finding should be interpreted cautiously because of the very small sample size.

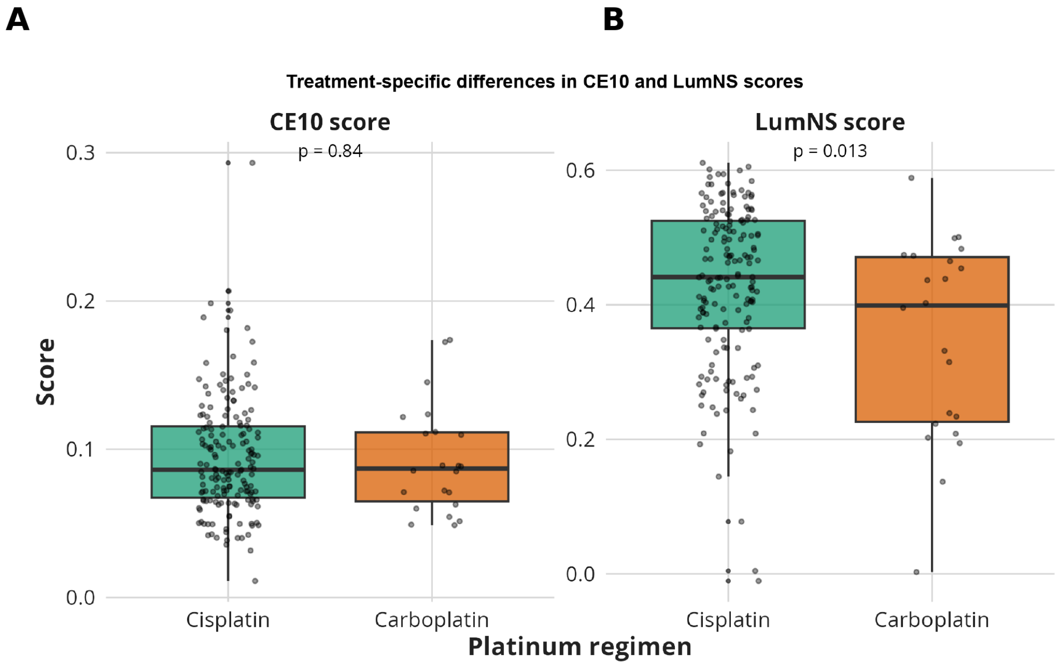

Supplementary Figure 10. Differences in immune cell infiltration and molecular subtypes scores between cisplatin- and carboplatin-treated patients. This figure illustrates the differences in Ecotype CE10 scores (A) and Luminal Non-Specified (LumNS) molecular subtype (B) score between the two treatment groups. Boxplots display the median (center line), interquartile range (box), and whiskers extending to 1.5× the interquartile range; individual data points are overlaid. Statistical comparisons used the Wilcoxon rank-sum test (P-values shown on each panel).

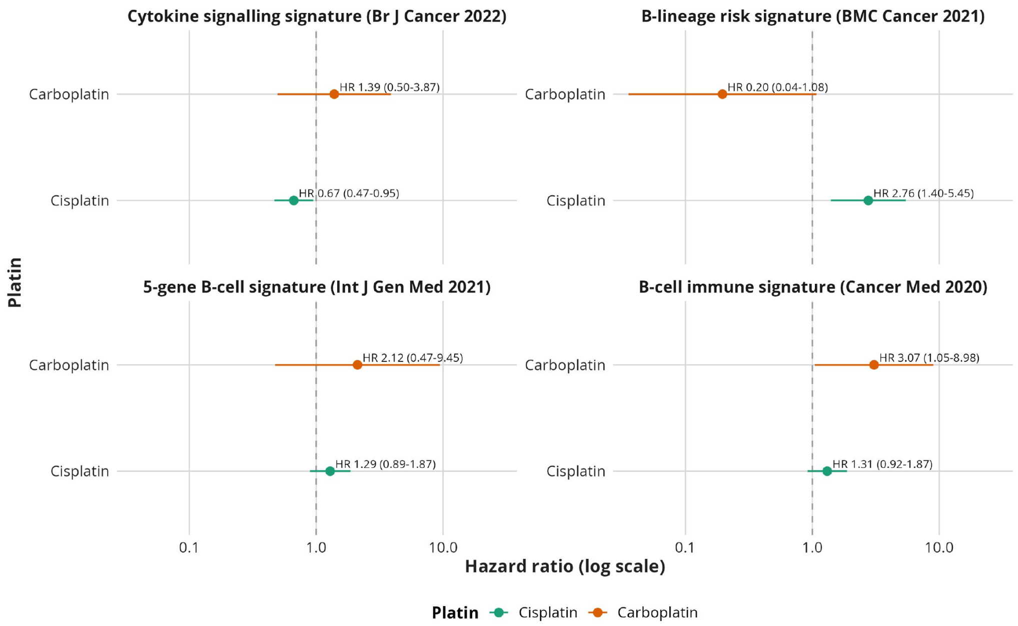

Supplementary Figure 11. Association of B-cell-related gene signatures with overall survival in cisplatin- versus carboplatin-treated patients. Forest plots show unadjusted Cox proportional hazards models for each B-cell-related gene signature in cisplatin-treated and carboplatin-treated subgroups. The numbers in parentheses next to each subgroup label indicate the number of patients (sample size, n) and the number of events (deaths) contributing to the corresponding hazard ratio (HR) estimate. Horizontal whiskers indicate the 95% confidence interval (95% CI) of the HR (not the standard deviation), and the central diamond marks the pooled HR estimate. P values from the Q-test for subgroup differences (cisplatin vs. carboplatin) are reported on each panel. CI = confidence interval; HR = hazard ratio; OS = overall survival.

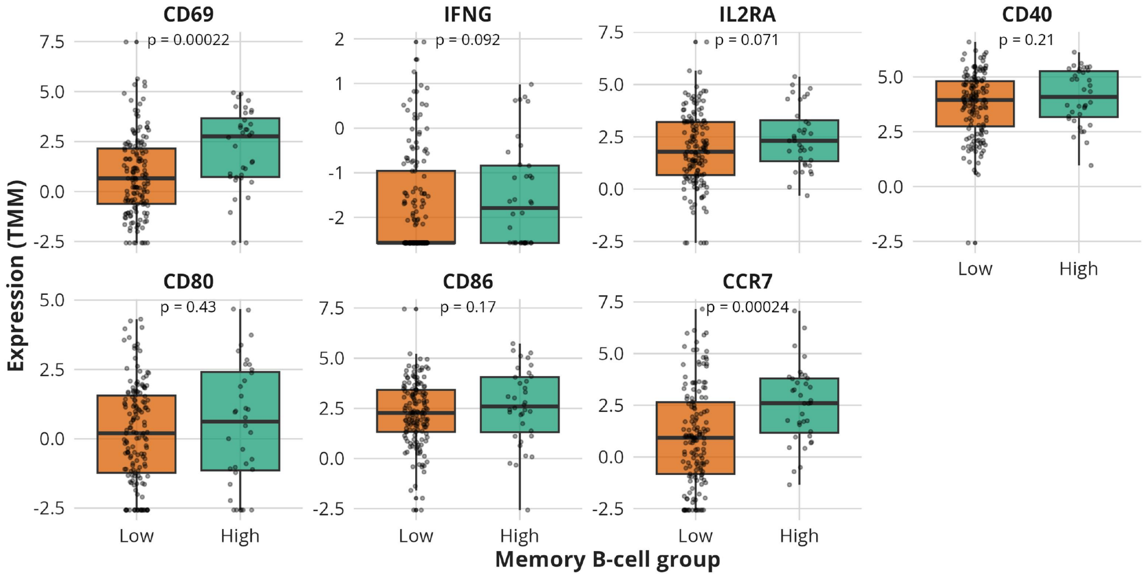

Supplementary Figure 12. Differential expression of immune activation markers in tumors with high versus low memory B-cell infiltration. Boxplots display the expression levels of T-cell activation markers (CD69, IFNG, IL2RA) and dendritic-cell activation markers (CD40, CD80, CD86, CCR7) in tumors stratified by high versus low memory B-cell infiltration. Box plots display the median (center line), the interquartile range (box: 25th–75th percentiles), and whiskers extending to 1.5 times the interquartile range; individual data points are overlaid. Statistical comparisons used the Wilcoxon rank-sum test, and the corresponding P values are displayed on each panel. Significant upregulation of CD69 (P = 0.00022) and CCR7 (P = 0.00024) was observed in tumors with high memory B-cell infiltration. Trend-level increases in IFNG (P = 0.092), IL2RA (P = 0.071), and CD86 (P = 0.17) suggest enhanced immune activation in memory B-cell-high tumors.

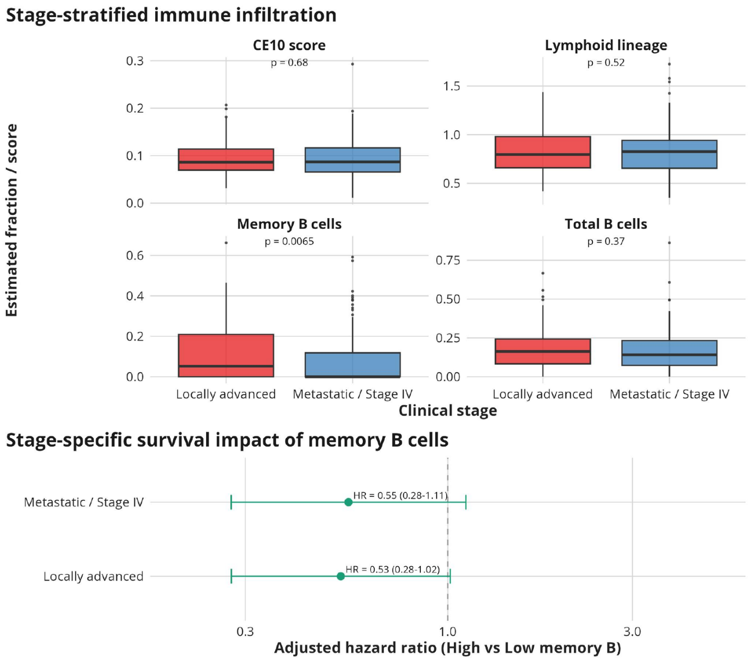

Supplementary Figure 13. Stage-stratified analysis of memory B-cell infiltration and overall survival in cisplatin-treated patients. (A) Distribution of memory B-cell fractions by disease stage (Wilcoxon p = 0.007). (B–C) Adjusted Cox regression estimates for locally advanced (HR = 0.53; 95% CI: 0.28–1.02; p = 0.056) and metastatic (HR = 0.55; 95% CI: 0.28–1.11; p = 0.097) subgroups. Cisplatin-treated patients only (n = 167).

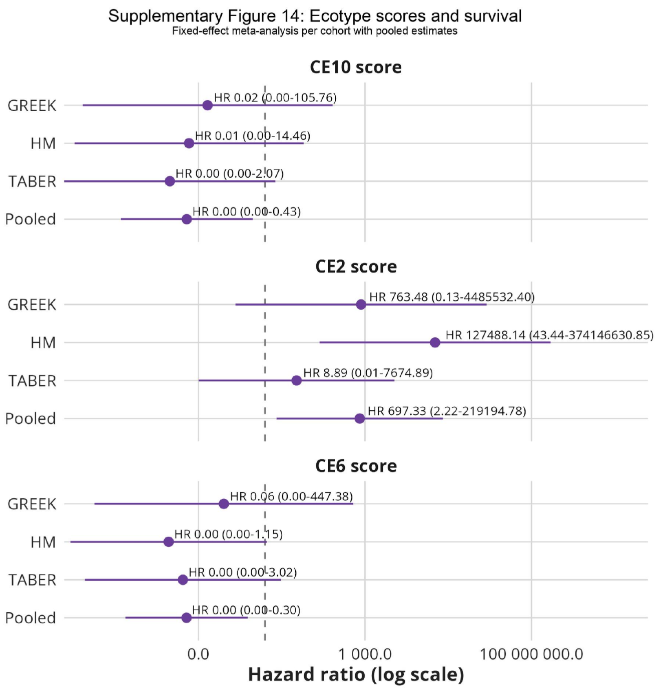

Supplementary Figure 14. Association between tumor ecotypes and overall survival. This figure presents hazard ratios (HRs) and 95% confidence intervals (CIs) for Ecotypes CE2, CE6, and CE10 derived from EcoTyper analysis, indicating their significant associations with overall survival.

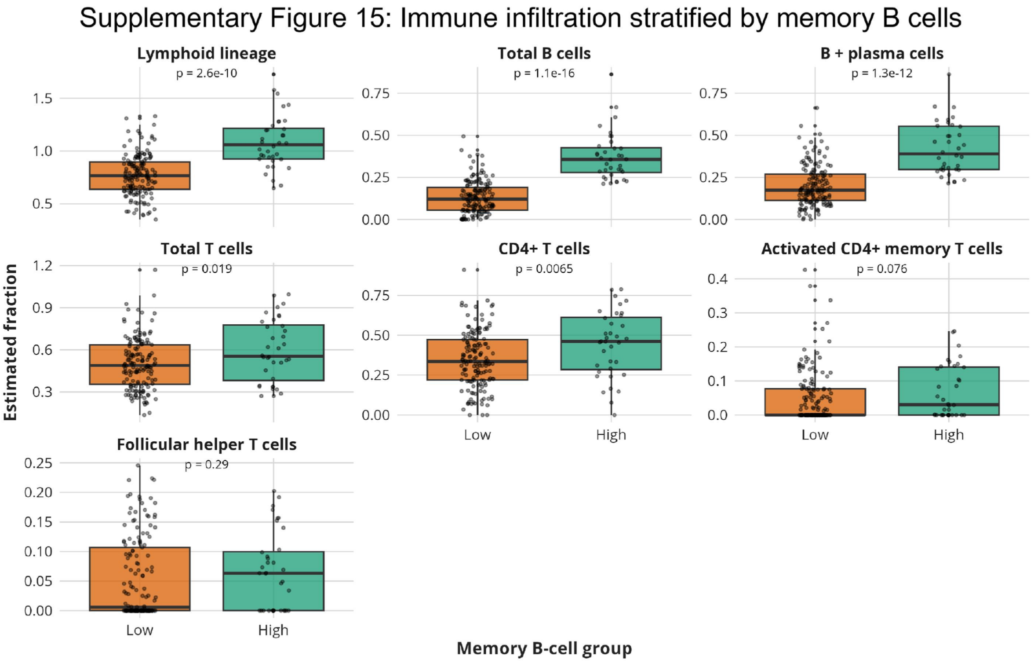

Supplementary Figure 15. Comparison of immune cell infiltration levels in tumors with high versus low memory B-cell infiltration. The figure shows boxplots of estimated immune cell proportions. Box plots display median (center line), interquartile range (box), and whiskers extending to 1.5 times the interquartile range. P-values were calculated using the Wilcoxon rank-sum test. Boxplots of estimated immune cell proportions, demonstrating increased lymphoid infiltration, particularly $CD4^+$ T cells, in tumors with high memory B-cell infiltration.

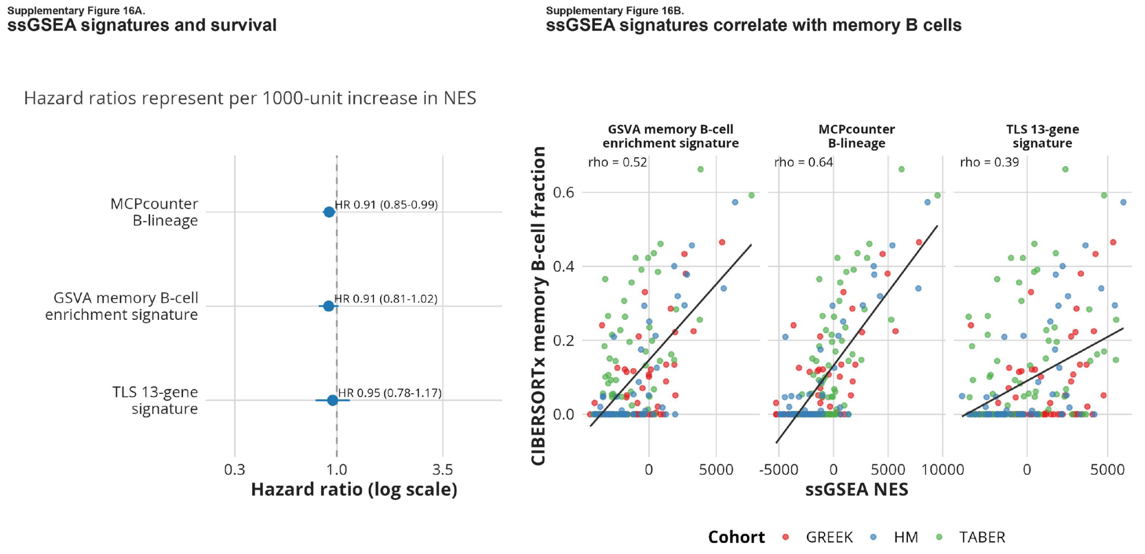

Supplementary Figure 16. Validation of CIBERSORTx memory B-cell fraction estimates using single-sample gene set enrichment analysis (ssGSEA) enrichment scores. (A) Scatter plots showing the Spearman rank correlations between CIBERSORTx memory B-cell fractions (x-axis) and ssGSEA normalized enrichment scores (y-axis) for the MCPcounter B-lineage signature, a custom memory B-cell panel, and the 13-gene tertiary lymphoid structure (TLS) signature across all patients (n = 189). (B) Density distributions of the same ssGSEA enrichment scores. The numbers in parentheses on each scatter plot denote the Spearman rank correlation coefficient (ρ) and the corresponding P value: MCPcounter B-lineage (ρ = 0.64, P < 0.001), custom memory B-cell panel (ρ = 0.52, P < 0.001), and 13-gene TLS signature (ρ = 0.39, P < 0.001). TLS = tertiary lymphoid structures.

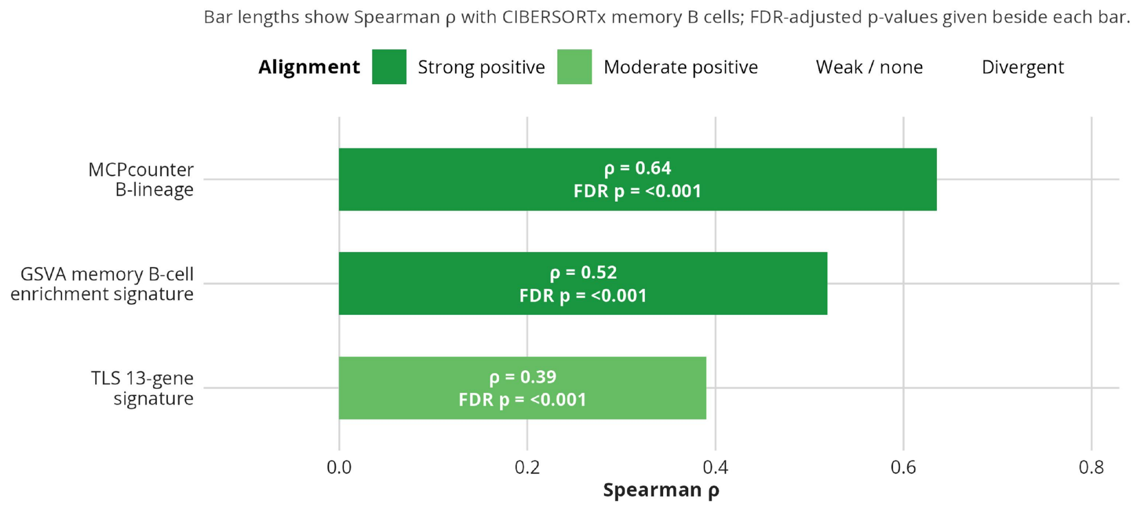

Supplementary Figure 17. Summary of Spearman rank correlations between CIBERSORTx memory B-cell fractions and ssGSEA normalized enrichment scores for the MCPcounter B-lineage, custom memory B-cell, and 13-gene TLS signatures. Bar lengths indicate correlation coefficients, and labels report FDR-adjusted significance.

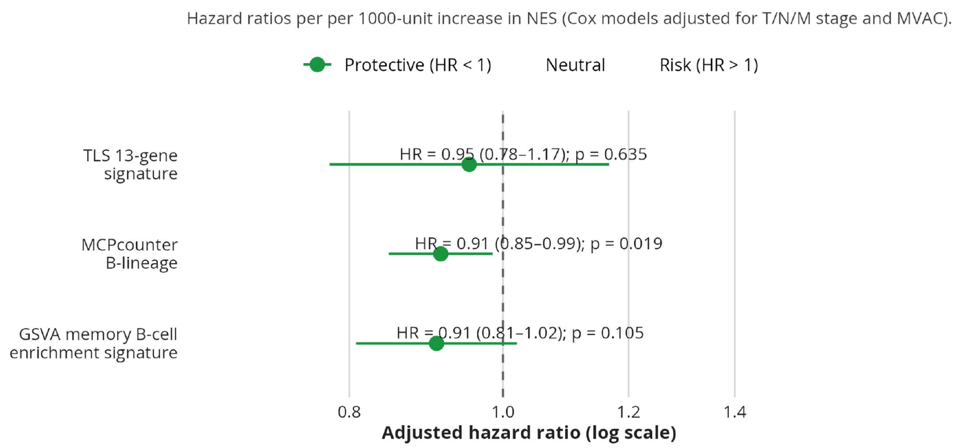

Supplementary Figure 18. Adjusted Cox proportional hazards models for ssGSEA-derived signature scores and overall survival. Higher MCPcounter B-lineage scores were associated with improved overall survival (adjusted HR = 0.91; 95% CI: 0.85–0.99; p = 0.019), whereas the custom memory B-cell and TLS signatures were not statistically significant.
